# The intrinsic defects of T cells impact the efficacy of CAR-T therapy in patients with diffuse large B-cell lymphoma

**DOI:** 10.1038/s41408-023-00958-9

**Published:** 2023-12-14

**Authors:** Jinrong Zhao, Chong Wei, Shuqing Wang, Yan Zhang, Wei Wang, Danqing Zhao, Zi Wang, Zhipeng Zhou, Jing Bai, Wei Zhang, Daobin Zhou

**Affiliations:** 1 Department of Hematology, Peking Union Medical College Hospital, Peking Union Medical College, Chinese Academy of Medical Sciences, Beijing, 100730 China; 2 Department of Hematology, Guangzhou First People’s Hospital, Guangzhou, 510180 China; 3 GenePlus-Beijing Institute, Beijing, 102206 China

**Keywords:** Cancer epigenetics, B-cell lymphoma

## Abstract

CAR-T cell therapy did not achieve the desired efficacy in some patients with diffuse large B-cell lymphoma (DLBCL). We conducted single-cell RNA and TCR sequencing as well as methylation chip profiling of peripheral blood samples in DLBCL patients. Patients who achieved complete remission (CR) showed an upward trend in T-cell levels, especially CD8-effector T cells. The responders exhibited T-cell clone expansion, more active T-cell transformation, and frequent cell communication. Highly expressed genes in the CR group were enriched in functions like leukocyte-mediated cytotoxicity and activation of immune response, while the non-CR group was enriched in pathways related to DNA damage and P53-mediated intrinsic apoptotic. More differentially methylated probes (DMPs) were identified in the baseline of the non-CR group (779 vs 350). GSEA analysis revealed that the genes annotated by DMPs were associated with cellular immune functions in T cells, including the generation of chemokines, leukocyte-mediated cytotoxicity, and cell-killing functions. The genes with low expression in the non-CR group exhibited a high methylation status. There is heterogeneity in the cellular, molecular, and epigenetic characteristics of host T cells in patients with different clinical outcomes. Intrinsic defects in T cells are important factors leading to poor efficacy of CAR-T therapy.

## Introduction

CAR-T therapy is a promising treatment for relapsed/refractory diffuse large B-cell lymphoma (R/R DLBCL). The therapy involves genetically modifying patients’ T cells to express chimeric antigen receptors (CARs) that can recognize and target cancer cells [1]. In DLBCL, CAR-T therapy has shown high response rates and long-term remission in patients who failed to respond to traditional chemotherapy [2, 3].

Despite its success in DLBCL patients, CAR-T therapy has faced challenges, such as its high cost, partial response or treatment failure in some patients, and potential serious side effects like cytokine release syndrome (CRS) and Immune effector cell-associated neurotoxicity syndrome (ICANS) [4, 5]. Early identification of patients who will respond to treatment and personalized treatment based on individual characteristics will be beneficial.

In the context of CAR-T cell therapy, T-cell functionality is crucial for the recognition and elimination of tumor cells. However, CAR-T cells have been shown to exhibit poor functionality in some cases, resulting in decreased efficacy and poor clinical outcomes [6, 7]. Therefore, future efforts in CAR-T cell therapy should focus on improving T-cell functionality to increase the overall efficacy and success of this treatment approach.

Therefore, we used single-cell sequencing (scRNA-seq, scTCR-seq) and Illumina Methylation EPIC 850k Beadchip to detect peripheral blood of R/R DLBCL patients before and after CD19 CAR-T cell infusion, in order to identify T cell clone dynamics, transcription programs, and methylation pattern characteristics associated with clinical outcomes.

## Methods

### Patients

Our study recruited 16 patients with relapsed/refractory DLBCL who received anti-CD19 CAR-T cell therapy at Peking Union Medical College Hospital from October 2021 to December 2022. The clinical characteristics of all patients are shown in Table 1. Among them, peripheral blood samples were collected from 5 patients before and after CAR-T cell infusion (day 14 and day 28) for single-cell analysis, and CD3 + T cells from peripheral blood samples were collected from 13 patients before CAR-T cell infusion for methylation chip array testing. Prior to treatment, the pathology slides of the patients were reviewed and the diagnosis was confirmed by three pathology experts according to the 5th edition of the World Health Organization classification of lymphoid hematopoietic tumors [8]. Based on the efficacy assessment one month after the patients received anti-CD19 CAR-T cell infusion, they were divided into two groups: the complete remission (CR) group and non-complete remission (non-CR) group. The study was approved by the Ethics Committee of Peking Union Medical College Hospital, Chinese Academy of Medical Sciences, and complied with the Helsinki Declaration. All patients signed informed consent before receiving treatment.

**Table 1:** Clinical characteristics of diffuse large B-cell lymphoma patients treated with CAR-T therapy.

ID	sex	age	Ann Arbor Stage	IPI	COO	ASCT	CAR-T	Prior lines	CRS Grade	ICANS Grade	Single-cell seq	Methylation Microarray	Clinical Outcomes
1	M	43	IVB	2	Non-GCB	-	Relma-cel	2	1	-	√	√	Non-CR
2	M	64	IVA	3	Non-GCB	-	Axi-cel	2	3	-	√	-	Non-CR
3	M	63	IIA	1	GCB	-	Axi-cel	2	2	-	√	√	CR
4	F	61	IIB	5	Non-GCB	-	Axi-cel	2	3	-	√	-	CR
5	F	58	IVB	4	Non-GCB	-	Relma-cel	3	1	-	√	-	CR
6	M	56	IIIB	3	GCB	-	Axi-cel	2	1	1	-	√	Non-CR
7	M	58	IVB	2	GCB	-	Relma-cel	2	2	1	-	√	Non-CR
8	F	61	IIIA	3	Non-GCB	-	Axi-cel	4	1	-	-	√	CR
9	M	61	IIA	1	Non-GCB	-	Axi-cel	2	1	-	-	√	CR
10	M	59	IVB	4	Non-GCB	-	Axi-cel	1	2	3	-	√	CR
11	M	57	IVA	3	Non-GCB	-	Relma-cel	4	-	-	-	√	CR
12	F	55	IIIA	2	Non-GCB	-	Axi-cel	2	1	-	-	√	CR
13	F	62	IVB	3	Non-GCB	-	Relma-cel	2	-	-	-	√	CR
14	M	47	IVA	2	Non-GCB	√	Relma-cel	1	-	-	-	√	CR
15	M	64	IVB	5	Non-GCB	-	Axi-cel	2	2	-	-	√	CR
16	F	54	IVB	2	Non-GCB	-	Relma-cel	4	-	-	-	√	CR

*F* female, *M* male, *COO* cell of origin, *ASCT* Autologous Stem Cell Transplan, *CRS* Cytokine release syndrome, *ICANS* Immune effector cell-associated neurotoxicity syndrome, *Relma-cel* Relmacabtagene autoleucel, *Axi-cel* axicabtagene ciloleucel.

### Single-cell RNA sequencing and bioinformatics analysis

Single-cell cDNA libraries were prepared using Chromium Single Cell 5′Library & Gel Bead Kit (10x Genomics). All procedures were performed according to the manufacturer’s instructions. The libraries were sequenced on a DNBSEQ-T7R platform (BGI, Shenzhen, China) with 150 bp paired-end read. CellRanger software (10x Genomics) was used to analyze the sequencing data and produced gene expression information for each cell. For quality control, cells with >10% reads mapping to mitochondria, or with <200 genes or <800 UMI counts indicated low quality, and were removed from the analysis. Then we used the Doublet Finder R package to identified and removed the doublets. Finally, we acquired 95,469 cells in total with an average of 6365 cells per each sample (range, 2800~9002). On average, 2006 genes and 6034 unique transcripts per cell were obtained. To remove batch effect, we used the integration methods in Seurat package (v4.0.2) to assemble multiple scRNA-seq samples into an integrated and unbatched dataset.

### Single-cell T-cell receptor (TCR) repertoire sequencing and data processing

Single-cell TCR VDJ libraries were prepared using Chromium Single Cell V(D)J Enrichment Kit (10× Genomics) according to the manufacturer’s instructions. Briefly, to obtain enrichment products, barcoded cDNA was applied to amplify full-length V, D, and J-gene segments using Chromium Single Cell V(D)J Enrichment Kit. Enriched product was measured by D5000 DNA Screen Tape analysis and Qubit dsDNA HS Assay Kit, and then used for library construction. Single-cell V(D)J enriched libraries were pooled for sequencing on DNBSEQ-T7R platform (BGI, Shenzhen, China) with 150 bp paired-end read. CellRanger VDJ (10× Genomics) was applied for TCR reconstruction and paired TCR clonotype calling. The CDR3 motif was located and the productivity was determined for each single cell. For quality control, TCR clones with low-confident, non-productive and UMIs <2 were removed. To integrate the scTCR data with the scRNA data, the TCR-based analysis was performed only for cells that were identified as T cells and cells with identical TCR β chains were defined as a T-cell clone. Finally, 36,861 cells with 15,470 clones were identified.

### Determination of TCR repertoire clonality and cell state transition

The clonality of TCR repertoire was quantified as follows [9]:

$${\rm{Clonality}}\,=\frac{\left(1-\mathop{\sum }\limits_{{\it{i}}=1}^{{\it{s}}}\left(\frac{{{\it{n}}}_{{\rm{i}}}}{{\it{N}}}\mathrm{ln}\frac{{{\it{n}}}_{{\it{i}}}}{{\it{N}}}\right)\right)}{\mathrm{ln}{\it{S}}}$$

In this formula, *n*_*i*_ is the clonal size of the ith clonotype, *S* is the total number of clonotypes, and *N* is the total number of TCR sequences analyzed. We identified shared motifs in the CDR3 sequence and used the overlapping cell fraction to evaluate cell state transition as reported previously [10].

### Dimension reduction and unsupervised clustering

We used the Seurat R package (v4.0.2) to perform dimension reduction and unsupervised clustering. The expression matrix was first normalized using the functions *NormalizeData* and *ScaleData*. Then principal component analysis (PCA) was applied to the normalized expression matrix with highly variable genes identified by *FindVariableGenes* function. The first 20 PCs were used for unsupervised clustering analysis with the resolution set to 2, yielding a total of 42 cell clusters. Further dimension reduction was performed by Uniform Manifold Approximation and Projection (UMAP) analysis using *RunUMAP* function in Seurat. *DimPlot* function was applied to visualize the clustering results.

### Cell type annotation and cluster marker identification

After dimension reduction and projection of cells into two-dimensional space by UMAP, cells clustered together according to canonical features. We then use the *FindAllMarkers* function in Seurat to find markers for each of the identified clusters with the following cutoff: |log2 fold change|≥0.25 and *P* value < 0.05 (Wilcoxon test). Clusters were annotated based on the expression of canonical genes of particular cell types. Clusters that expressed no canonical cell type markers were identified as low-quality cells, and expressed two or more canonical cell type markers were identified as doublet cells. Both low-quality and doublet cells were excluded from further analyses.

### Subclustering of major cell types

For each major cell type, cells were extracted from the count dataset first. Next, we use the *NormalizeData* and *ScaleData* function to normalized expression matrix. PCA was applied to the normalized expression matrix with highly variable genes identified by *FindVariableGenes* function. We then use *RunHarmony* function in harmony R package to remove batch effect. At last, dimension reduction was performed by UMAP analysis using *RunUMAP* function in Seurat.

### Cell-cell interaction analysis

To analyze cell-cell interactions between different cell types, we used CellPhoneDB (v2.1.1) [11] to identify significant ligand-receptor pairs in CR and non-CR group, respectively. Briefly, the cell type-specific receptor-ligand interactions between different cell types were identified based on the specific expression of a receptor in one cell type and a ligand in another cell type. We assessed the number of significant interactions in CR and non-CR group, and explored specific interactions.

### SCENIC analysis

Single-Cell rEgulatory Network Inference and Clustering (SCENIC) analysis [12] was performed to reveal the difference in gene regulatory network between CR and non-CR CD8-effector T cell. We use the pySCENIC (v0.12.1), a lightning-fast python implementation of the SCENIC pipeline, to performed SCENIC analysis.

### Whole-genome methylation assay

Peripheral blood mononuclear cells were separated using the Ficoll method, and CD3 + T cells were selected using magnetic beads (Maitaini, Germany). The sorting efficiency was >95% as assessed by flow cytometry. Subsequently, DNA was extracted using a genomic DNA extraction kit (Tiangen, China), and the DNA concentration was measured using a Nanodrop 2000 spectrophotometer, with the OD260/280 ratio being controlled between 1.6–1.8. DNA integrity was evaluated using agarose gel electrophoresis. Whole-genome methylation detection was performed by Shanghai OE Biotech Co., Ltd.

### Statistical analysis

A *p* value of <0.05 was considered statistically significant. Data analysis was performed using GraphPad Prism 9.0 and R version 4.1.3. Categorical variables were presented as frequencies and percentages. Continuous variables were presented as mean ± standard deviation.

## Results

### Clinical characteristics

The clinical characteristics of all patients are shown in Table 1. The median age was 58.5 years (range 43–64 years), including 10 cases (62.5%) of males and 6 cases (37.5%) of females. Three patients (18.8%) were diagnosed with Ann Arbor Stage II, 3 patients (18.8%) were Stage III, and 10 patients (62.5%) were Stage IV. Three patients (18.8%) were sourced from germinal centres, while the remaining 13 cases (71.2%) were sourced from activated B cells. Seven patients (43.8%) had an International Prognostic Index (IPI) ≤ 2. Only 1 patient (6.3%) had undergone autologous hematopoietic stem cell transplantation before CAR-T cell infusion. Ten patients (62.5%) experienced grade 1–2 CRS after infusion, and 3 patients (18.8%) experienced ICANS. Among the 16 patients included in the study, 7 received Relmacabtagene autoleucel as the CD19 CAR-T infusion product, while the other 9 received Axicabtagene ciloleucel. Of the 4 patients with poor efficacy, 2 received Relmacabtagene autoleucel infusion.

### Single-cell transcriptomic profiling before and after CAR-T treatment

**Fig. 1:**
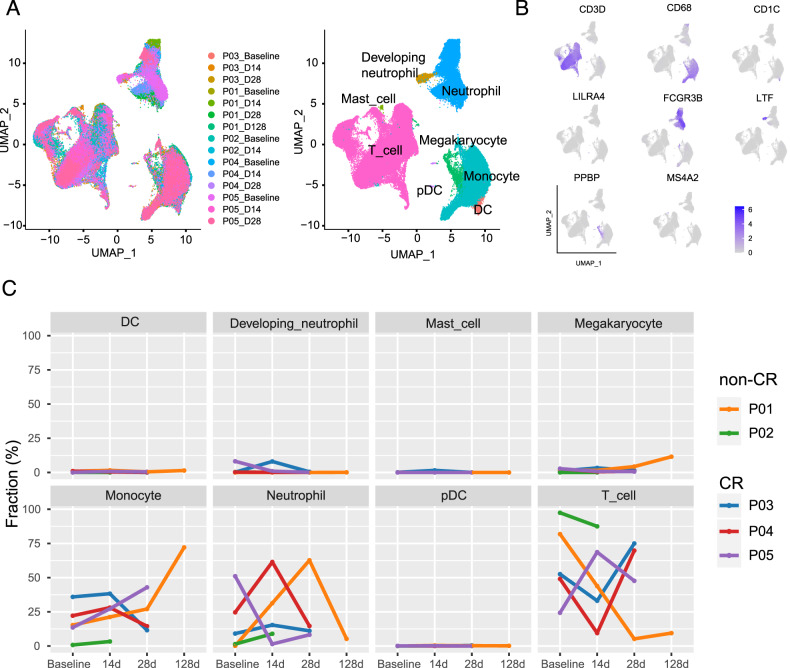
Single-cell transcriptional profiles of peripheral blood samples from DLBCL patients before and after CAR-T therapy. **A** Annotation of cell clusters, color-coded according to the corresponding patient, sampling timepoint, and cell subpopulation. **B** Key marker genes for each cell cluster, including T cells (CD3D), myeloid cells (CD68), DC cells (CD1C), pDC cells (LILRA4), neutrophils (FCGR3B), developing neutrophils (LTF), megakaryocytes (PPBP), and mast cells (MS4A2). **C** Changes in the proportion of different cell types over time.

Of the 5 patients, 3 achieved complete remission after treatment. One patient was evaluated as partial remission (PR) at 1 month after infusion, but was later evaluated as CR at 3 months. Unfortunately, another patient experienced progressive disease and died, so no peripheral blood sample was obtained at d28. Single-cell sequencing was carried out on 15 PBMC samples from 5 patients before and after CAR-T therapy, yielding a total of 95,469 valid cells, with 52,090 cells from the CR group and 43,379 cells from the non-CR group. UMAP clustering produced 42 clusters (Supplementary Fig. 1A, Supplementary Table 1), which were further annotated into 8 cell types, including T cells (CD3D, CD3G, CD2), myeloid cells (CD68), DC cells (CD1C, CD1E), pDC cells (LILRA4), neutrophils (FCGR3B, CSF3R), immature neutrophils (LTF), megakaryocytes (PPBP), and mast cells (MS4A2) (Fig. 1A, B, Supplementary Fig. 1B). More than half of the cells were T cells (54%), followed by monocytes (24%) and neutrophils (17%) (Supplementary Fig. 1C). We compared the proportion of each cell type in the CR and non-CR groups before and after treatment and found that the proportion of T cells increased in the CR group while it decreased in the non-CR group (Fig. 1C, Supplementary Fig. 1D). There were no significant differences in other cell types between the two groups.

### CD8+ effector T cells exhibit enhanced cytotoxicity in CR patients, while expressing apoptosis program in non-CR patients

**Fig. 2:**
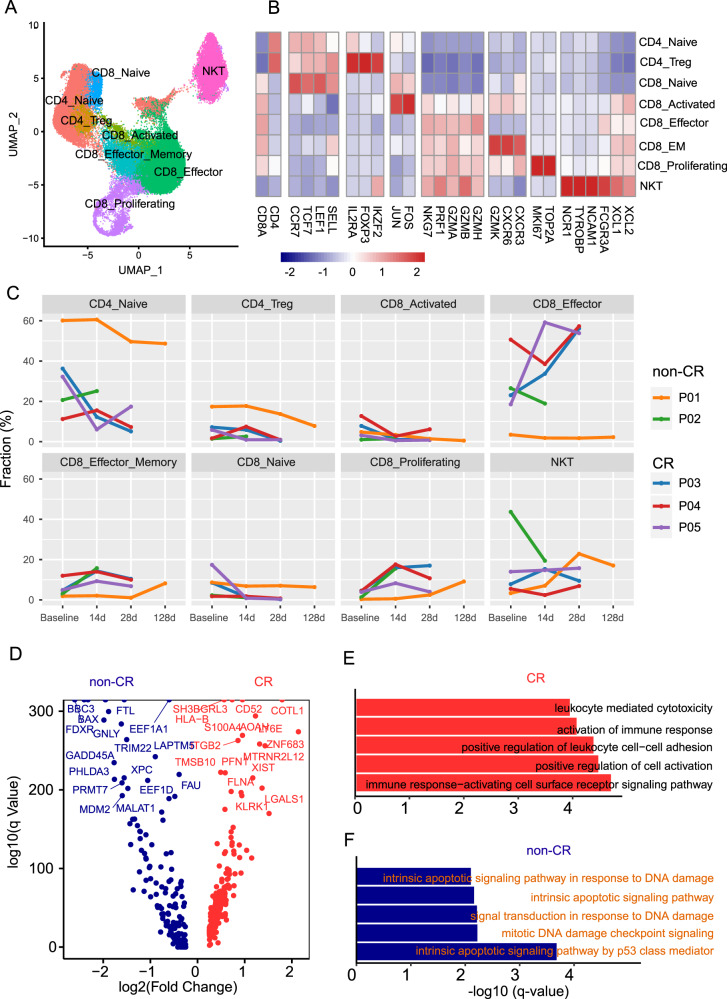
Single-cell transcriptional profiles of T cells. **A** Subgroup classification of T cells, with each color representing a different subgroup. **B** Expression of marker genes in each T cell subgroup. **C** Changes in the proportion of T cell subgroups over time in two patient groups. **D** Volcano plot showing differential gene expression in CD8-Effector T cells between the two patient groups at baseline. Red indicates genes with higher expression in the CR group, while blue indicates genes with higher expression in the non-CR group. **E** GO enrichment results for highly expressed genes in the CR group, showing the top 5 enrichment results. **F** KEGG enrichment results for highly expressed genes in the non-CR group, showing the top 5 enrichment results.

Eight T-cell subgroups were identified, including NK T (NCR1), 5 CD8 subtypes, namely CD8-Naive (IL7R, CCR7), CD8-effector_Memory (GZMA, GZMK), CD8-proliferating (TOP2A, MKI67), CD8-effector (GZMA, PRF1), and CD8-Activated (JUN, FOS), and 2 CD4 subtypes, namely CD4-Naïve (IL7R, CCR7) and CD4-Treg (FOXP3) (Fig. 2A, B). Analysis of the proportion of each subtype before and after treatment revealed an increase in the proportion of CD8-effector cells after treatment in the CR group, while the non-CR group showed a decrease (Fig. 2C). Therefore, the CD8-effector subtype was further investigated by differential gene analysis between the CR and non-CR groups (Fig. 2D). The highly expressed genes in the CR group were enriched in functions such as leukocyte-mediated cytotoxicity, activation of immune response, and positive regulation of leukocyte cell-cell adhesion. On the other hand, the non-CR group was enriched in pathways related to DNA damage, P53-mediated intrinsic apoptosis, and other related pathways (Fig. 2E, F). Protein interaction analysis confirmed these findings, with CD38, FCGR3A, CD52, and CD69 being the central proteins in cell activation and killing in the CR group (Supplementary Fig. 2), while GADD45A, PCNA, BBC3, and BAX were involved in cell apoptosis and cell cycle arrest were central in the non-CR group (Supplementary Fig. 3). The higher correlation level of these genes in non-CR group supported the hypothesis that DNA damage may have occurred in T cells, leading to a series of activations in the cell cycle arrest, DNA damage repair, and cell apoptosis pathways (Supplementary Fig. 4A). High expression levels of the crucial genes BAX and BBC3 in these pathways were monitored using TCR data, revealing a decrease in these cells after treatment, indicating cell apoptosis (Supplementary Fig. 4B, C). Differential analysis was also carried out on CD8 effector at day 14 after treatment, yielding similar results (Supplementary Fig. 4D, E). Furthermore, CD8-effector transcription factor activation was analyzed before and after treatment, uncovering the involvement of various transcription factors, such as NFATC2 and EOMES (Supplementary Fig. 5A–C).

### T cell expansion was associated with better clinical outcomes of CAR-T treatment

**Fig. 3:**
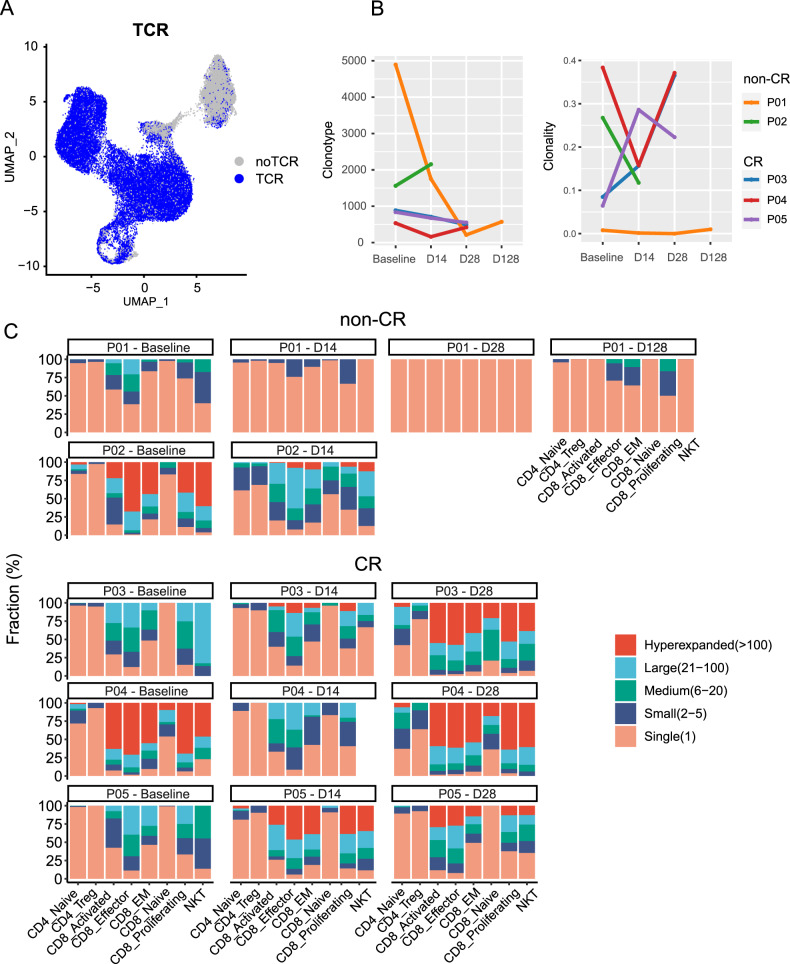
Single-cell TCR sequencing atlas of peripheral blood samples from DLBCL patients. **A** scTCR coverage plot. **B** Number of clone types and oligoclonality changes. **C** Clone structure of each subtype.

Single-cell TCR sequencing was performed on 15 samples, and shared information between scRNA and scTCR was matched. 75% of T cells could detect corresponding TCR sequences, with most NKT cells not detected (Fig. 3A, Supplementary Table 2). The number of clone types and oligoclonality were calculated for each sample, with non-CR groups having more clone types and showing a decreasing trend in oligoclonality after treatment (Fig. 3B). Furthermore, we analyzed the clone structure of each T cell subtype in each sample, dividing T cell clones into five categories: hyperexpanded (>100 T cells), large (21–100), medium (6–20), small (2–5), and single (1). Longitudinal analysis of the clone structure revealed that in the CR group, cytotoxic cells such as CD8-effector, CD8-activated, CD8-EM, and CD8-proliferating expanded after treatment, while in the non-CR group, cytotoxic cells decreased (Fig. 3C). We further analyzed the top 10 clone types in each sample and found that patients with good efficacy had clone expansion, mainly in CD8-effector cells, while patients with poor efficacy had clone reduction (Supplementary Fig. 6). An analysis of the treatment process revealed that patients with better efficacy had more active T cell type transformations (Supplementary Fig. 7).

### Interactions between cells are associated with the clinical outcomes of CAR-T therapy

**Fig. 4:**
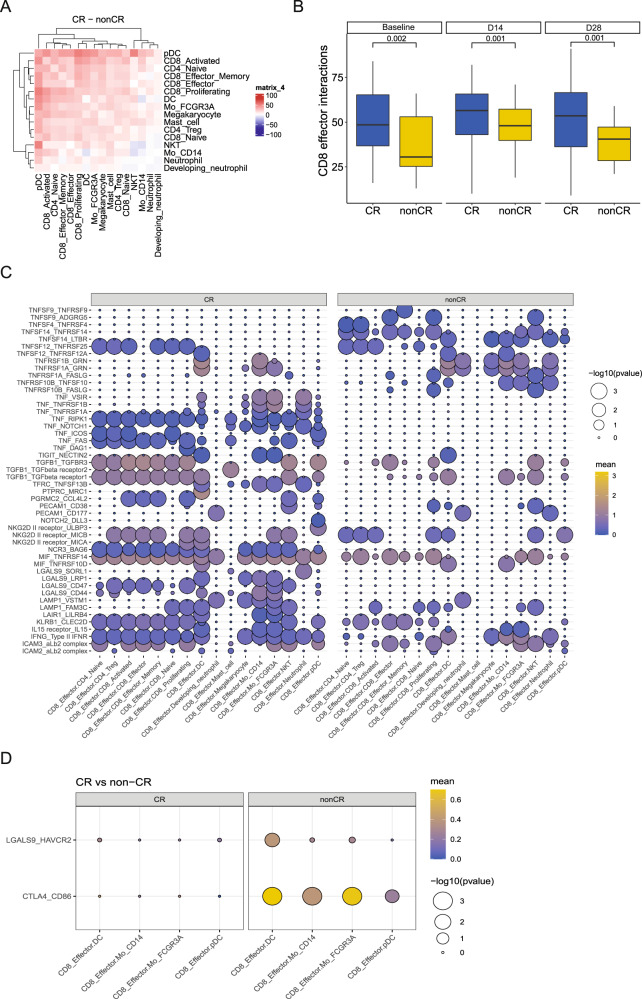
Analysis of cell communication in peripheral blood samples of DLBCL patients. **A** Comparison of cell communication at baseline between two groups of patients. **B** Comparison of cell interaction of CD8-effector T cells at baseline, D14 and D28. **C** Cell interaction of CD8-effector T cells and other cells in two groups of patients. **D** Interaction related to immune suppression in two groups of patients.

In order to understand the impact of different cellular interactions on the effectiveness of CAR-T therapy, we conducted cell communication analysis. The results showed that the baseline cell communication in the CR group was more frequent compared to the non-CR group (Fig. 4A). We further focused on CD8-effector T cells and found that their interactions were more pronounced in the CR group, both before and after treatment (Fig. 4B). Specifically, CD8-effector T cells in the CR group exhibited more interactions with other cells involving tumor necrosis factors, TGFβ, and NK cell killing (Fig. 4C). Additionally, we observed immune-inhibitory interactions in the non-CR group after treatment (Fig. 4D).

### The methylation landscape of host T cells influences the clinical outcomes of patients

Currently, the association between epigenetic changes and immune cell function, as well as the occurrence and progression of lymphoma, is gaining increasing attention. Subsequently, we explored the relationship between the methylation landscape of host T cells before CAR-T cell infusion and the clinical outcomes of patients.

**Fig. 5:**
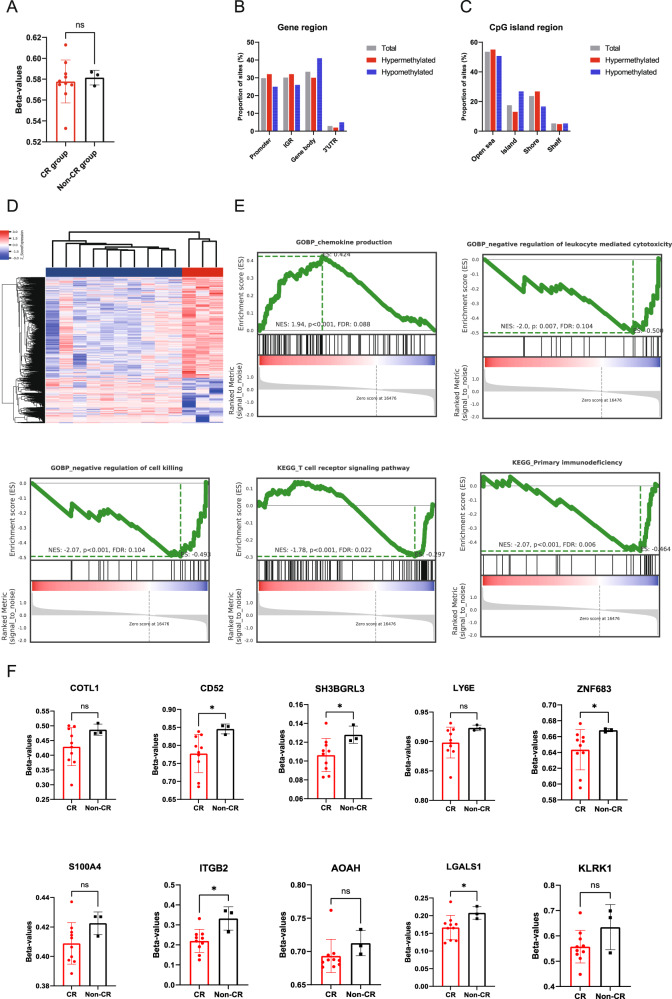
Epigenetic profiles of peripheral blood T cells in DLBCL patients. Box plots showing the average methylation levels of all sites in both groups (**A**). Distribution of differentially methylated sites in gene regions (**B**) and CpG islands (**C**) in both groups. Cluster heatmap depicting differentially methylated sites (**D**). In the cluster analysis heatmap, blue represents low methylation levels, and red represents high methylation levels. GSEA plot of differentially methylated sites in both groups (**E**), with the red portion indicating upregulated gene expression in the CR group, the blue portion indicating high expression in the non-CR group, and the gray portion indicating the signal-to-noise ratio of each gene. Methylation levels of the Top 10 high-expressing genes in the CR group (**F**). ES enrichment score, NES normalized enrichment score, FDR false discovery rate, ns not significant; *, *P* < 0.05.

The average baseline methylation level in the non-CR group was slightly higher compared to the CR group, but the difference was not statistically significant (0.582 ± 0.007 vs 0.578 ± 0.021, *P* = 0.811) as shown in Fig. 5A. A total of 1129 differentially methylated probes (DMPs), including 779 hypermethylated sites and 350 hypomethylated sites, were identified between the baseline levels of the two groups (non-CR group vs CR group). The heatmap of clustering analysis revealed significant differences in the methylation status between the two groups (Fig. 5D). These differential methylation sites were primarily located in the promoter region (29.8%), intergenic regions (30.1%), and gene bodies (33.4%). Analysis of the relative positions of DMPs to CpG showed that the majority of differential sites were distributed in the open sea region (53.5%), and additionally, 26.8% of hypomethylated sites were in the island region (Fig. 5B, C). The GSEA results indicated that the genes annotated by these differentially methylated sites were associated with the activation of cellular immune functions in T cells, including the production of chemokines, leukocyte-mediated cytotoxicity, and cell killing functions. In terms of KEGG analysis, these genes were closely related to the T cell receptor signaling pathway and primary immunodeficiency (Fig. 5E). Next, we analyzed the methylation status of the Top 10 high-expressing genes found in the CR group in the previous transcriptome analysis, with all genes in the non-CR group having higher beta-values than those in the CR group (Fig. 5F).

## Discussion

The rapid development of adoptive cell therapies, such as CAR-T cells, have greatly changed the current tumor treatment strategies, but their successful application is largely limited by T cell dysfunction or exhaustion [13–15]. Due to sustained stimulation from tumor antigens, the host T cells in the tumor microenvironment, which serve as the source material for CAR-T cells, are often in a dysfunctional state [16, 17]. This greatly impacts the clinical outcomes of patients. Therefore, it is crucial to improve the function of host T cells before CAR-T cell manufacturing and to identify the functional differences in host T cells early on in order to predict the therapeutic efficacy for patients [18].

This study aims to explore the impact of inherent defects in T cells on the suboptimal therapeutic efficacy of CAR-T treatment in DLBCL patients. Compared to non-CR patients, CR group patients have more close interplay and coordination between CD8-effector T cells and other cells. In addition, the results of single-cell TCR sequencing also suggest clonal expansion of T cells and more active T cell type conversion in patients with good treatment response. Previously, researchers have revealed significant differences in the transcriptome analysis of CAR-T infusion products from patients with different treatment responses in processes such as effector differentiation, glycolysis, exhaustion, and cell apoptosis [19]. Aberrant expression of host T cell genes may lead to defects in T cell function, severely limiting the ability of CAR-T cells to fully clear tumor cells. For example, COTL1, an actin-binding protein [20], regulates actin cytoskeleton in immune cells by interacting with α and β-actin as well as F-actin, serving as a T cell activator in the immune system [21]. Studies have shown that it can inhibit tumor growth by activating IL-24/PERP and suppressing atypical TGFβ signaling [22]. SH3 domain binding glutamic acid-rich protein like 3 (SH3BGRL3) plays an important regulatory role in various biological processes such as cell signal transduction, proliferation, and differentiation, but the underlying molecular mechanisms of these functions are not yet clear [23]. LY6E (Lymphocyte Antigen 6E) is a cell surface membrane protein that plays an important role in the immune system. It can enhance the activity of natural killer cells (NK cells) and T cells, and promote the infiltration and cytotoxicity of immune cells against tumor cells [24–26]. ZNF683 (Zinc Finger Protein 683) is a domain protein involved in the activation and differentiation of T cells, and it can also promote the differentiation and functional regulation of specific immune cells [27]. However, further in vivo and in vitro experiments are needed to confirm the specific molecular mechanisms of these core genes and their roles.

Correcting these intrinsic defects in T cells is crucial for improving the effectiveness of CAR-T cell therapy. A range of strategies has been explored to enhance the efficacy of adoptive T cells, including the pre-application of ibrutinib [28], coupling with anti-tumor cytokines [29], and the use of immune checkpoint inhibitors such as PD-1 or PD-L1 antibodies [30]. Epigenetic mechanisms play a significant role in the activation, differentiation, and effector function of immune cells [31, 32]. In this study, the average methylation level of host T cells in the non-CR group was relatively higher compared to the CR group, and there were more highly methylated sites, with distinct methylation profiles between the two groups. Lowering the methylation level is beneficial for enhancing the expression of anti-cancer genes. Researchers have found through whole-genome bisulfite sequencing (WGBS) of exhausted CD8 + T cells that T cells gradually acquire an inheritable de novo methylation program mediated by DNMT3A, which limits T cell expansion capacity and clonal diversity during immunotherapy. Pre-treatment with DNA-demethylating drugs such as decitabine before immunotherapy enhances the proliferation ability of dysfunctional CD8 + T cells [33]. Studies have revealed that treating CAR T cells with decitabine in vitro and in vivo enhances their anti-tumor activity, cytokine production, and proliferation ability [34]. Furthermore, evidence suggests that demethylating drugs can increase the immunogenicity of tumor cells, thereby enhancing T cell-mediated cytotoxicity [35, 36]. In this study, differentially downregulated genes in the non-CR group were found to be in a highly methylated state, with signaling pathways such as cell killing showing a downregulation trend. Since previous studies have reported that DNA methylation of certain genes such as LY6E [37], S100A4 [38], ITGB2 [39], LGALS1 [40, 41], and IL2RG [42] can downregulate gene expression, we speculate that the application of demethylating drugs may help regulate the expression of related immune genes, improve T lymphocyte dysfunction, and increase the immunogenicity of tumor cells, thereby enhancing the efficacy of subsequent CAR-T cell therapy. However, these assumptions need to be further validated through in vivo and in vitro experiments. DNA hydroxymethylase TET2 catalyzes the conversion of 5-methylcytosine (5mC) to 5-hydroxymethylcytosine (5hmC) to change the DNA methylation status [43]. Interestingly, knocking out TET2 can alter the differentiation status and proliferation ability of T lymphocytes, effectively enhancing the efficacy of CAR-T cell therapy [44]. Pre-application of TET2 inhibitors to alter the epigenetic status of host T lymphocytes and CAR-T cells before cell isolation is a direction worth exploring.

There are some limitations in this study. The key genes obtained in this study have not been further validated. Additionally, due to the small sample size, it may not be applicable to all patients.

In summary, we reported the interplay and collaboration between host T cells, especially CD8-effector T cells, and other immune cells in patients with different clinical outcomes of CAR-T therapy. Longitudinal analysis showed that positive T-cell clonal expansion and phenotypic transformation were associated with better efficacy. The intrinsic genetic defects of T cells may be an important factor contributing to suboptimal efficacy in DLBCL patients, and modifying the epigenetic status of host T cells to restore normal gene expression and address the intrinsic limitations of host T cells may improve the function of CAR-T cells. This may be a promising direction for future CAR-T cell therapy.

### Supplementary information

supplementary materials

## Data Availability

The datasets used and/or analyzed during the current study are available from the corresponding author on reasonable request.
