# Supplementary material for: The intrinsic defects of T cells impact the efficacy of CAR-T therapy in patients with diffuse large B-cell lymphoma
Source: Blood Cancer J. 2023 Dec 14;13(1):186. doi: 10.1038/s41408-023-00958-9 (PMC10721638; doi:10.1038/s41408-023-00958-9)
Supplement: Supplementary file 1 — supplementary materials [file 41408_2023_958_MOESM1_ESM.pdf]

Supplementary Figure 1. Single-cell transcriptome profiles of DLBCL before and after CAR-T therapy. A) Identification of 42 clusters. B) Expression patterns of marker genes in each cell cluster. C) Total cell counts across the quality-controlled cell clusters. D) Bar plot depicting changes in the proportions of each cell cluster.

Supplementary Figure 2. Protein interaction analysis of highly expressed genes in the CR group.

Supplementary Figure 3. Protein interaction analysis of highly expressed genes in the non-CR group.

Supplementary Figure 4. A) Correlation of apoptosis-related genes in CD8-Effector T cells between the CR and non-CR groups. B) Monitoring the expression changes of cells with high expression of BBC3 in the non-CR group at baseline and day 14. C) Monitoring the expression changes of cells with high expression of BAX in the non-CR group at baseline and day 14. D) Volcano plot depicting differentially expressed genes in CD8-Effector T cells between the two groups on day 14 post-infusion. Red represents differentially expressed genes with high expression in the CR group, while blue represents differentially expressed genes with high expression in the non-CR group. E) KEGG enrichment results of highly expressed genes in the CR group. F) KEGG enrichment results of highly expressed genes in the non-CR group.

Supplementary Figure 5. Activation status of transcription factors in CD8-effector T cells. The figure shows the differentially expressed transcription factors between the CR and non-CR groups at baseline (A), day 14 (B), and day 28 (C).

Supplementary Figure 6. Top 10 clones for each sample.

Supplementary Figure 7. Clone conversion status for each sample.

Supplementary Table 1. Top 20 marker genes for all cells.

Supplementary Table 2. Share of TCRs for all samples.

Supplementary Table 3. Clone types and clonality for all samples.

Figure 1

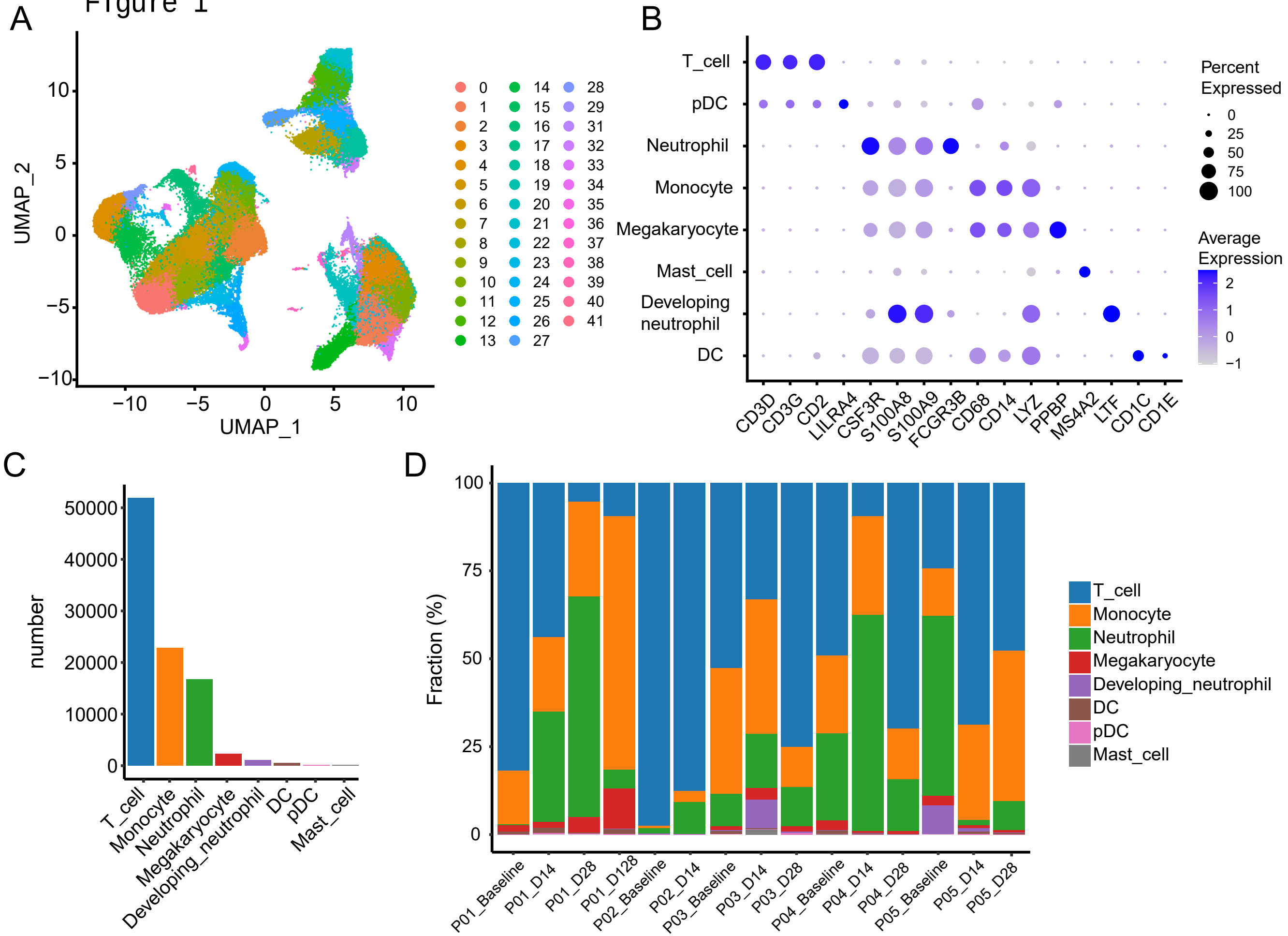
A
UMAP_2
UMAP_1
10
5
0
−5
−10
0 1 2 3 4 5 6 7 8 9 10 11 12 13 14 15 16 17 18 19 20 21 22 23 24 25 26 27 28 29 31 32 33 34 35 36 37 38 39 40 41
B
T_cell
pDC
Neutrophil
Monocyte
Megakaryocyte
Mast_cell
Developing neutrophil
DC
CD3D CD3G CD2 LILRA4 CSF3R S100A8 S100A9 FCGR3B CD68 CD14 LYZ PPBP MS4A2 LTF CD1C CD1E
Percent Expressed
0 25 50 75 100
Average Expression
2 1 0 −1
C
number
50000 40000 30000 20000 10000 0
T_cell Monocyte Neutrophil Megakaryocyte Developing_neutrophil DC pDC Mast_cell
D
Fraction (%)
100 75 50 25 0
P01_Baseline P01_D14 P01_D28 P01_D128 P02_Baseline P02_D14 P03_Baseline P03_D14 P03_D28 P04_Baseline P04_D14 P04_D28 P05_Baseline P05_D14 P05_D28
T_cell
Monocyte
Neutrophil
Megakaryocyte
Developing_neutrophil
DC
pDC
Mast_cell

Figure 2

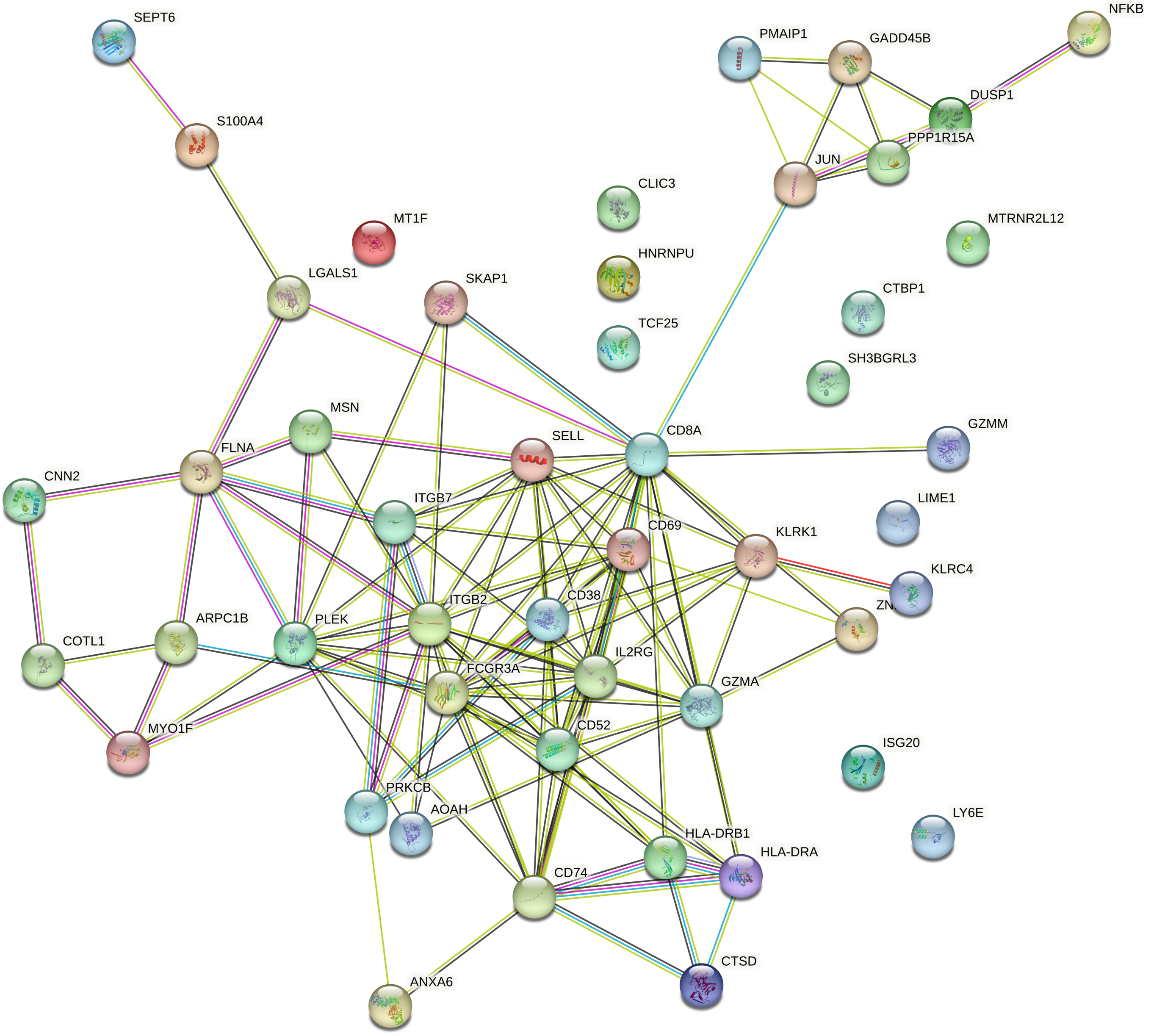
SEPT6
S100A4
MT1F
LGALS1
SKAP1
CLIC3
HNRNPU
TCF25
PMAIP1
GADD45B
NFKB
DUSP1
PPP1R15A
JUN
MTRNR2L12
CTBP1
SH3BGRL3
GZMM
LIME1
KLRC4
KLRK1
CD8A
SELL
MSN
FLNA
CNN2
ITGB7
CD69
ITGB2
CD38
ARPC1B
PLEK
COTL1
IL2RG
FCGR3A
GZMA
MYO1F
CD52
ISG20
LY6E
PRKCB
AOAH
HLA-DRB1
HLA-DRA
CD74
CTSD
ANXA6

Figure 3

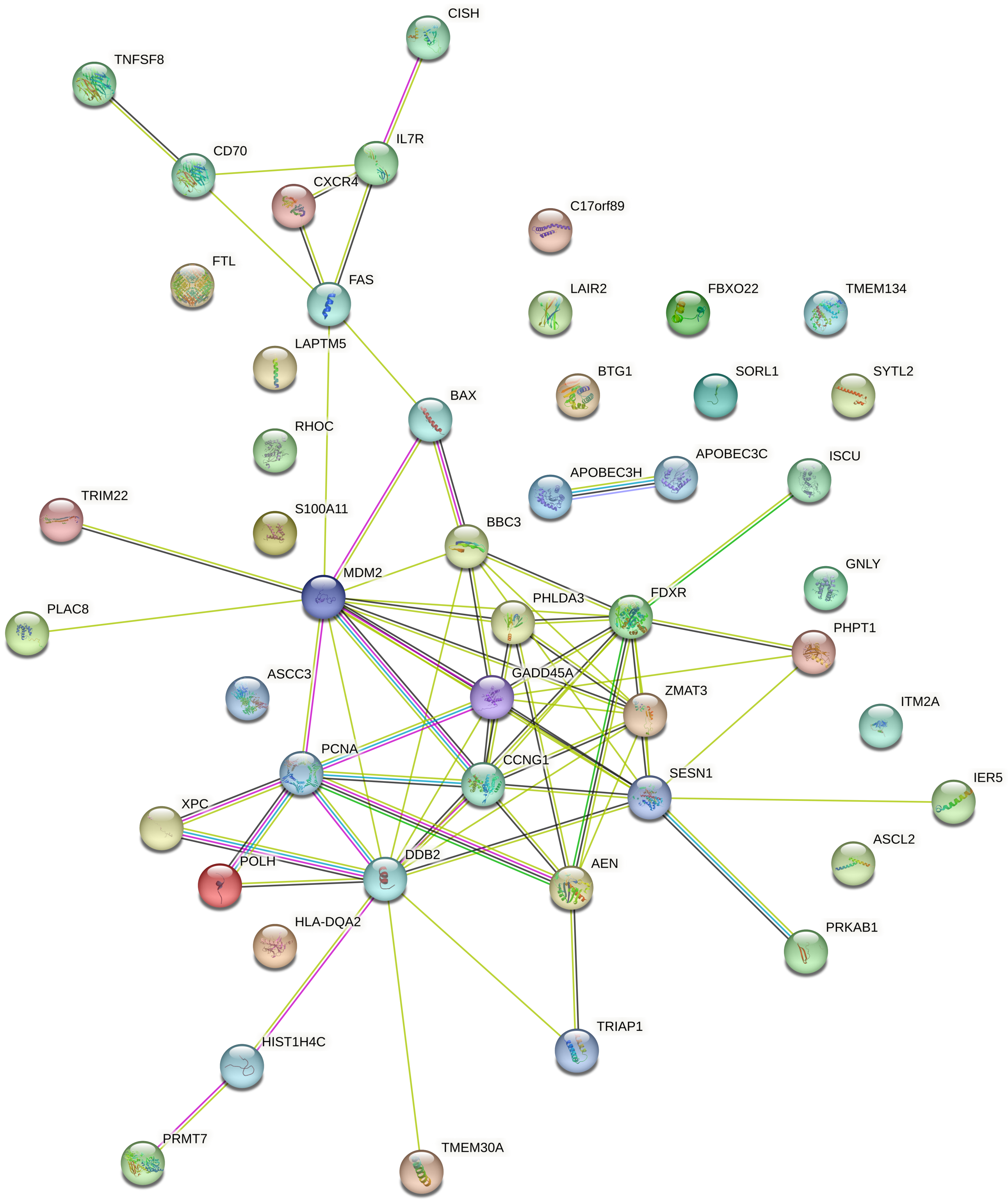
CISH
TNFSF8
CD70
IL7R
CXCR4
C17orf89
FTL
FAS
LAIR2
FBXO22
TMEM134
LAPTM5
BTG1
SORL1
SYTL2
BAX
RHOC
APOBEC3C
ISCU
APOBEC3H
TRIM22
S100A11
BBC3
MDM2
GNLY
PHLDA3
FDXR
PLAC8
PHPT1
GADD45A
ASCC3
ZMAT3
ITM2A
PCNA
CCNG1
SESN1
IER5
XPC
ASCL2
DDB2
POLH
AEN
HLA-DQA2
PRKAB1
TRIAP1
HIST1H4C
PRMT7
TMEM30A

Figure 4

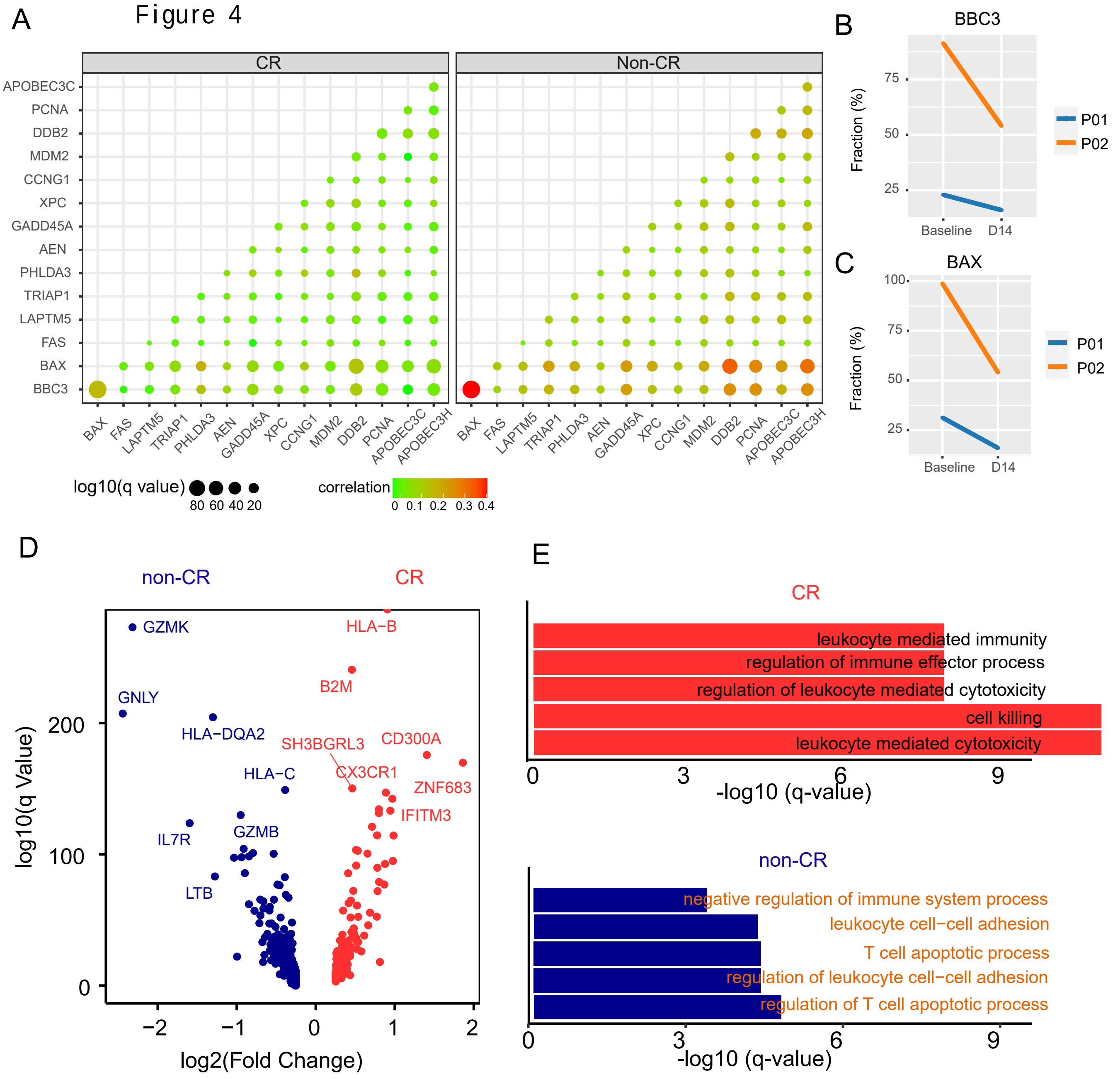

A
CR
Non-CR
APOBEC3C
PCNA
DDB2
MDM2
CCNG1
XPC
GADD45A
AEN
PHLDA3
TRIAP1
LAPTM5
FAS
BAX
BBC3
APOBEC3H
log10(q value)
80 60 40 20
correlation
0 0.1 0.2 0.3 0.4
B
BBC3
Fraction (%)
Baseline
D14
P01
P02
C
BAX
D
non-CR
CR
log10(q Value)
log2(Fold Change)
GZMK
GNLY
HLA−DQA2
HLA−C
IL7R
GZMB
LTB
HLA−B
B2M
SH3BGRL3
CD300A
CX3CR1
ZNF683
IFITM3
E
CR
leukocyte mediated immunity
regulation of immune effector process
regulation of leukocyte mediated cytotoxicity
cell killing
leukocyte mediated cytotoxicity
-log10 (q-value)
non-CR
negative regulation of immune system process
leukocyte cell−cell adhesion
T cell apoptotic process
regulation of leukocyte cell−cell adhesion
regulation of T cell apoptotic process

Figure 5

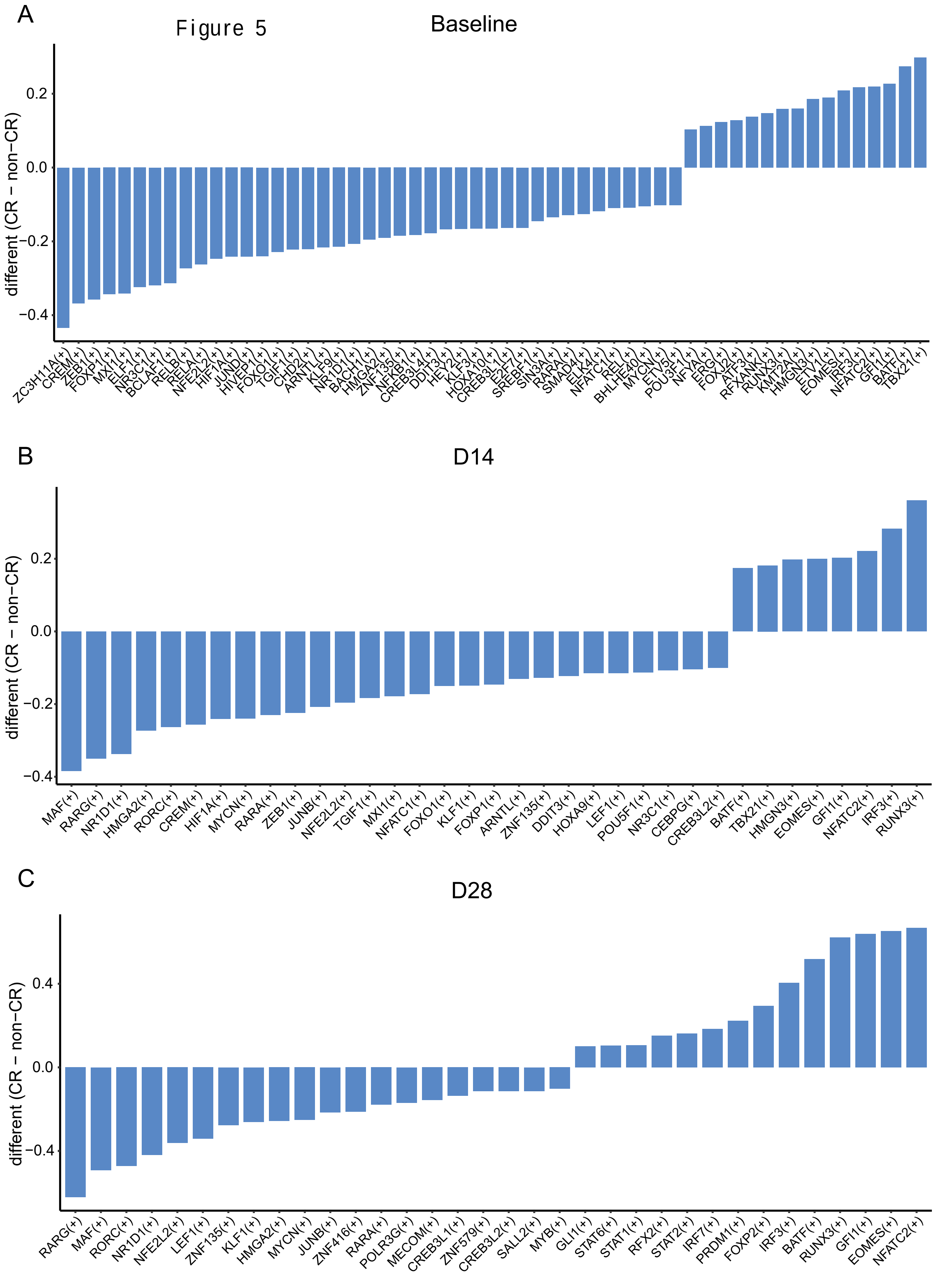
A
Baseline
different (CR − non−CR)
0.2
0.0
−0.2
−0.4
ZC3H11A(+) CREM(+) ZEB1(+) FOXP1(+) MXI1(+) ELF1(+) NR3C1(+) BCLAF1(+) RELB(+) RELA(+) NFE2L2(+) HIF1A(+) JUND(+) HIVEP1(+) FOXO1(+) TGIF1(+) CHD2(+) ARNTL(+) KLF9(+) NR1D1(+) BACH1(+) HMGA2(+) ZNF135(+) NFKB1(+) CREB3L4(+) DDIT3(+) HEY2(+) KLF3(+) HOXA10(+) CREB3L1(+) E2F7(+) SREBF1(+) SIN3A(+) RARA(+) SMAD4(+) ELK4(+) NFATC1(+) REL(+) BHLHE40(+) MYCN(+) ETV5(+) POU3F1(+) NFYA(+) ERG(+) FOXJ2(+) ATF3(+) RFXANK(+) RUNX3(+) KMT2A(+) HMGN3(+) ETV1(+) EOMES(+) IRF3(+) NFATC2(+) GFI1(+) BATF(+) TBX21(+)
B
D14
different (CR − non−CR)
0.2
0.0
−0.2
−0.4
MAF(+) RARG(+) NR1D1(+) HMGA2(+) RORC(+) CREM(+) HIF1A(+) MYCN(+) RARA(+) ZEB1(+) JUNB(+) NFE2L2(+) TGIF1(+) MXI1(+) NFATC1(+) FOXO1(+) KLF1(+) FOXP1(+) ARNTL(+) ZNF135(+) DDIT3(+) HOXA9(+) LEF1(+) POU5F1(+) NR3C1(+) CEBPG(+) CREB3L2(+) BATF(+) TBX21(+) HMGN3(+) EOMES(+) GFI1(+) NFATC2(+) IRF3(+) RUNX3(+)
C
D28
different (CR − non−CR)
0.4
0.0
−0.4
RARG(+) MAF(+) RORC(+) NR1D1(+) NFE2L2(+) LEF1(+) ZNF135(+) KLF1(+) HMGA2(+) MYCN(+) JUNB(+) ZNF416(+) RARA(+) POLR3G(+) MECOM(+) CREB3L1(+) ZNF579(+) CREB3L2(+) SALL2(+) MYB(+) GLI1(+) STAT6(+) STAT1(+) RFX2(+) STAT2(+) IRF7(+) PRDM1(+) FOXP2(+) IRF3(+) BATF(+) RUNX3(+) GFI1(+) EOMES(+) NFATC2(+)

Figure 6

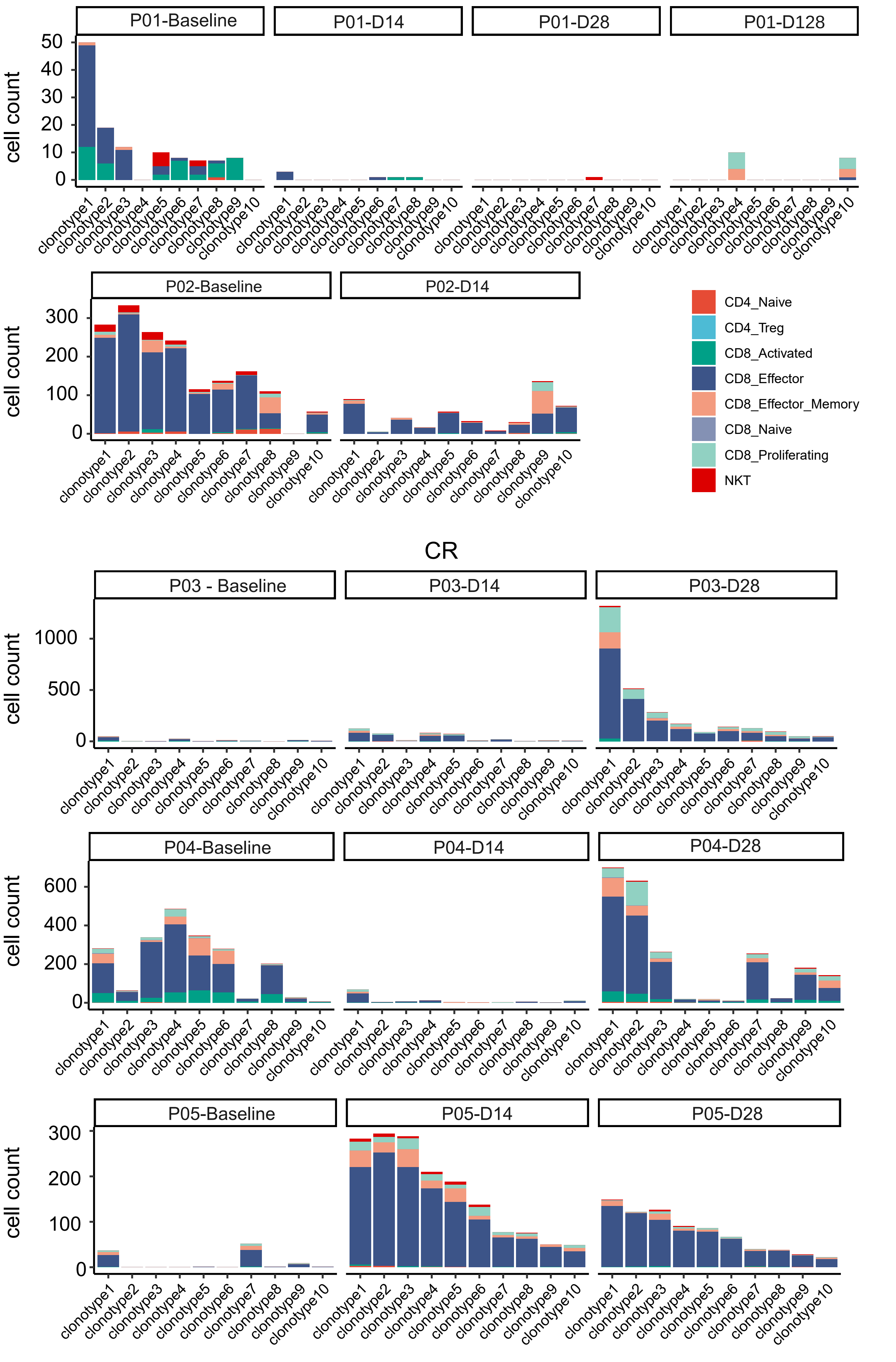

non-CR
P01-Baseline
P01-D14
P01-D28
P01-D128
P02-Baseline
P02-D14
cell count
clonotype1
clonotype2
clonotype3
clonotype4
clonotype5
clonotype6
clonotype7
clonotype8
clonotype9
clonotype10
CD4_Naive
CD4_Treg
CD8_Activated
CD8_Effector
CD8_Effector_Memory
CD8_Naive
CD8_Proliferating
NKT
CR
P03 - Baseline
P03-D14
P03-D28
P04-Baseline
P04-D14
P04-D28
P05-Baseline
P05-D14
P05-D28

Figure 7

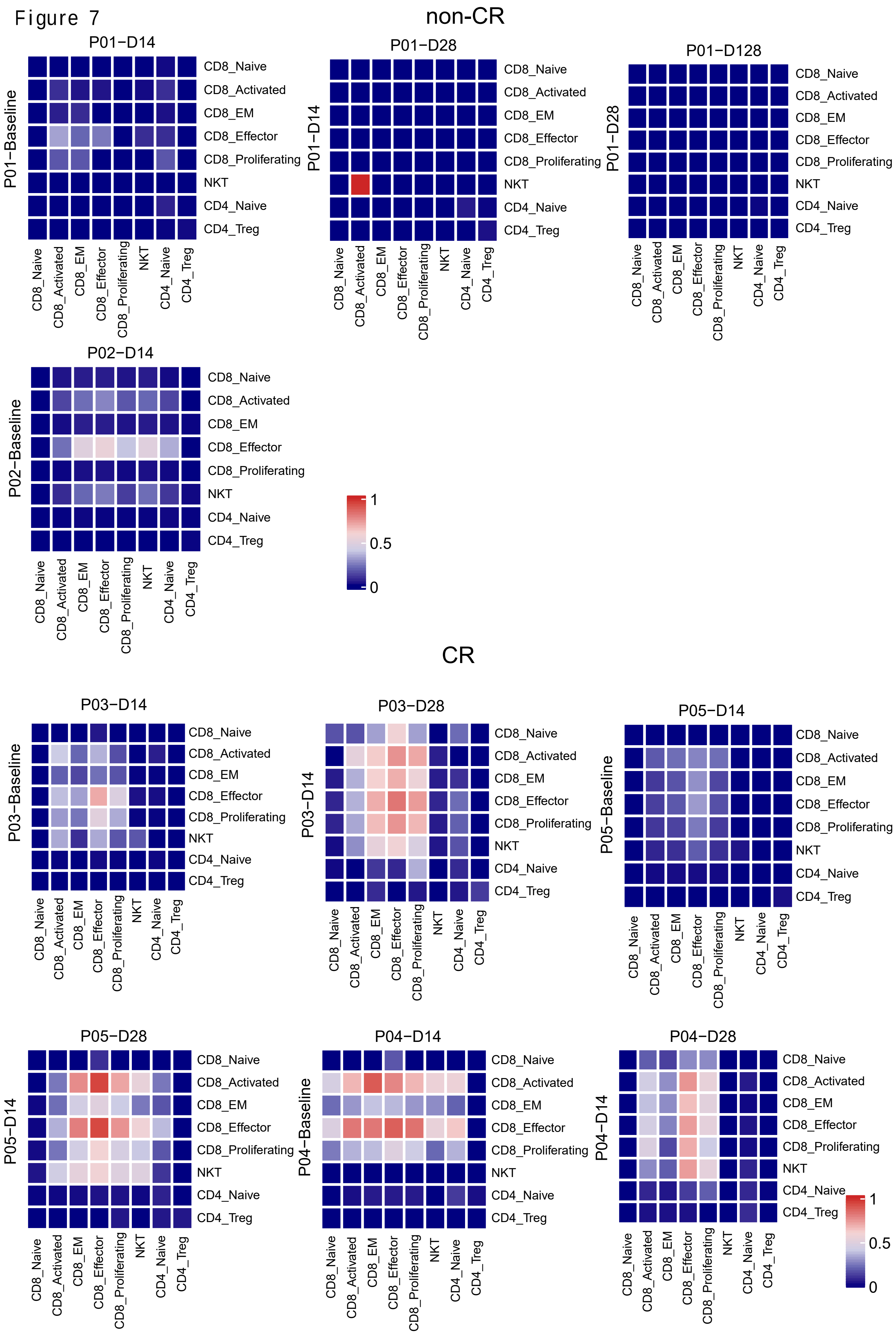
non-CR
P01−D14
P01−D28
P01−D128
P01−Baseline
P01−D14
P01−D28
P02−D14
P02−Baseline
CD8_Naive
CD8_Activated
CD8_EM
CD8_Effector
CD8_Proliferating
NKT
CD4_Naive
CD4_Treg
1
0.5
0
CR
P03−D14
P03−D28
P05−D14
P03−Baseline
P03−D14
P05−Baseline
P05−D28
P04−D14
P04−D28
P05−D14
P04−Baseline
P04−D14

Supp Table 1: Top 20 markers of all clusters

p_val	avg_log2F(	pct.1	pct.2	p_val_adj	cluster	gene
0	1.1914995	1	0.547	0	0	NKG7
0	1.1722104	0.999	0.372	0	0	GZMH
0	1.1105469	0.985	0.555	0	0	CD8A
0	1.0954195	0.984	0.581	0	0	CD8B
0	1.0300312	1	0.656	0	0	CCL5
0	1.0229862	0.983	0.361	0	0	FGFBP2
0	0.835159	0.999	0.744	0	0	IL32
0	0.8324507	0.992	0.527	0	0	TRBV11-2
0	0.8301729	0.98	0.636	0	0	CTSW
0	0.8214619	0.999	0.614	0	0	CST7
0	0.815586	0.986	0.695	0	0	CD3G
0	0.7559734	0.997	0.823	0	0	CD3D
0	0.7438658	0.865	0.305	0	0	GZMB
0	0.7252477	0.999	0.85	0	0	CD3E
0	0.7198345	0.969	0.729	0	0	GZMM
0	0.6473676	0.973	0.359	0	0	GZMA
0	0.6452737	0.977	0.646	0	0	ABHD17A
0	0.6245815	0.854	0.424	0	0	TBX21
0	0.6175169	0.907	0.634	0	0	C12orf75
0	0.6175035	0.968	0.743	0	0	LCK
0	1.2395	1	0.687	0	1	LYZ
0	1.1780898	0.999	0.732	0	1	HLA-DRA
0	1.1709245	1	0.517	0	1	IFI30
0	1.1587876	1	0.726	0	1	CST3
0	1.0541763	0.973	0.529	0	1	CPVL
0	1.0498864	0.986	0.414	0	1	GRN
0	1.0239428	0.999	0.535	0	1	FCN1
0	1.0116755	0.996	0.669	0	1	CD68
0	1.0084763	0.999	0.55	0	1	TYMP
0	0.9999964	0.997	0.659	0	1	LGALS3
0	0.9896483	0.998	0.775	0	1	HLA-DRB5
0	0.960737	1	0.806	0	1	HLA-DRB1
0	0.9575656	0.9	0.341	0	1	LGALS2
0	0.9489643	0.996	0.631	0	1	NPC2
0	0.9399979	1	0.744	0	1	CTSS
0	0.9360742	0.997	0.487	0	1	TNFAIP2
0	0.9349747	0.993	0.49	0	1	SERPINA1
0	0.928877	0.973	0.473	0	1	LRP1
0	0.9204507	0.95	0.448	0	1	VCAN
0	0.9097448	1	0.721	0	1	AIF1
0	0.8376868	0.987	0.97	0	2	IL7R
0	0.803408	0.97	0.749	0	2	LTB
0	0.6496229	0.988	0.801	0	2	KLF2
0	0.636217	0.926	0.89	0	2	CD5
0	0.6279175	0.92	0.726	0	2	ARID5B
0	0.6088873	0.816	0.711	0	2	ITM2A
0	0.6079974	0.91	0.845	0	2	RCAN3
0	0.5986921	0.926	0.844	0	2	SESN3

0	0.598376	0.877	0.901	0	2	MAL
0	0.5941979	0.991	0.852	0	2	CD3E
0	0.5823607	0.982	0.922	0	2	SPOCK2
0	0.5646418	0.872	0.818	0	2	TRAT1
0	0.5645335	0.982	0.947	0	2	LDHB
0	0.5488802	0.893	0.894	0	2	TCF7
0	0.5459692	0.946	0.9	0	2	ITK
0	0.544263	0.986	0.904	0	2	ETS1
0	0.5407182	0.896	0.889	0	2	CAMK4
0	0.5312614	0.991	0.981	0	2	PIK3IP1
0	0.5213762	0.936	0.854	0	2	SFXN1
0	0.5183683	0.918	0.913	0	2	RORA
0	1.1972819	1	0.688	0	3	LYZ
0	1.0881002	0.998	0.726	0	3	CST3
0	1.0334987	0.937	0.45	0	3	VCAN
0	1.0055044	0.991	0.519	0	3	IFI30
0	0.9350319	0.999	0.56	0	3	S100A8
0	0.9243216	0.999	0.597	0	3	S100A9
0	0.9068074	0.958	0.51	0	3	CSTA
0	0.9062426	0.978	0.537	0	3	FCN1
0	0.8662795	0.991	0.722	0	3	AIF1
0	0.8646245	0.986	0.767	0	3	SLC25A37
0	0.8623638	0.974	0.552	0	3	TYMP
0	0.8383525	0.998	0.7	0	3	TYROBP
0	0.8293184	0.96	0.491	0	3	TNFAIP2
0	0.8278325	0.997	0.744	0	3	CTSS
0	0.81506	0.976	0.671	0	3	CD68
0	0.8065337	0.963	0.601	0	3	CEBPD
0	0.8018669	0.95	0.497	0	3	CSF3R
0	0.7912247	0.938	0.444	0	3	S100A12
0	0.7831684	0.967	0.662	0	3	LGALS3
0	0.7824722	0.846	0.342	0	3	MS4A6A
0	1.5092221	0.96	0.526	0	4	GNLY
0	1.4723162	0.998	0.307	0	4	GZMB
0	1.3902911	1	0.554	0	4	NKG7
0	1.3268473	0.942	0.506	0	4	SPON2
0	1.3061917	0.995	0.641	0	4	CTSW
0	1.2734161	0.997	0.49	0	4	PRF1
0	1.2003501	0.956	0.373	0	4	FGFBP2
0	1.1868067	0.999	0.62	0	4	CST7
0	1.1822609	0.94	0.418	0	4	KLRD1
0	1.1805047	0.988	0.368	0	4	GZMA
0	1.1631849	0.978	0.291	0	4	S1PR5
0	1.1605944	0.992	0.662	0	4	CCL5
0	1.1319013	0.93	0.347	0	4	CCL4
0	1.0874766	0.859	0.261	0	4	CLIC3
0	0.9909718	0.884	0.786	0	4	KLRB1
0	0.9413779	0.852	0.309	0	4	KLRF1
0	0.9366225	0.995	0.859	0	4	CD7
0	0.9342273	0.958	0.404	0	4	FCGR3A
0	0.9187667	0.847	0.56	0	4	TRBC1

0	0.9184801	0.983	0.822	0	4	CD247
0	0.621431	0.991	0.853	0	5	CD3E
0	0.5714548	0.82	0.571	0	5	CD8A
0	0.5669309	0.99	0.663	0	5	CCL5
0	0.5183132	0.998	0.969	0	5	IL7R
0	0.4752761	0.963	0.828	0	5	CD3D
0	0.4582136	0.978	0.683	0	5	JUN
0	0.4301102	0.996	0.922	0	5	SPOCK2
0	0.4233237	0.91	0.799	0	5	PCED1B-AS1
0	0.409967	0.948	0.859	0	5	C12orf57
0	0.4060401	0.998	0.981	0	5	PIK3IP1
0	0.3937367	0.99	0.959	0	5	GAS5
0	0.3904393	0.86	0.79	0	5	LIME1
0	0.3875114	0.899	0.838	0	5	CD6
0	0.3873258	0.916	0.797	0	5	CD2
0	0.3860439	0.998	0.935	0	5	CXCR4
0	0.3772761	0.993	0.946	0	5	TLE5
0	0.3760827	0.998	0.978	0	5	NOP53
0	0.3714149	0.88	0.706	0	5	CD3G
3.82E-251	0.4933197	0.739	0.601	7.64E-248	5	CD8B
2.10E-193	0.374677	0.705	0.582	4.19E-190	5	LYAR
0	0.8694322	0.952	0.59	0	6	CD8B
0	0.8684803	0.996	0.383	0	6	GZMH
0	0.8584887	1	0.555	0	6	NKG7
0	0.84344	0.974	0.563	0	6	CD8A
0	0.7949655	0.973	0.373	0	6	FGFBP2
0	0.7838538	1	0.662	0	6	CCL5
0	0.6919089	0.998	0.535	0	6	TRBV11-2
0	0.6506153	0.998	0.853	0	6	CD3E
0	0.6168751	0.995	0.826	0	6	CD3D
0	0.5969442	0.964	0.701	0	6	CD3G
0	0.5611709	0.999	0.748	0	6	IL32
0	0.5596602	0.994	0.621	0	6	CST7
0	0.5121501	0.87	0.648	0	6	CTSW
0	0.5119225	0.783	0.536	0	6	GNLY
0	0.5084274	0.859	0.315	0	6	GZMB
0	0.4966069	0.933	0.798	0	6	PCED1B-AS1
0	0.491075	0.933	0.735	0	6	GZMM
0	0.4656976	0.949	0.748	0	6	LCK
0	0.459661	0.921	0.755	0	6	PTPRCAP
2.43E-242	0.4677456	0.662	0.259	4.87E-239	6	ZNF683
0	1.0190304	0.999	0.563	0	7	S100A8
0	0.9684342	0.985	0.446	0	7	S100A12
0	0.8834195	0.841	0.414	0	7	S100P
0	0.8035143	0.998	0.601	0	7	S100A9
0	0.7050331	0.845	0.383	0	7	PROK2
0	0.6727518	0.937	0.52	0	7	GCA
0	0.6427125	0.819	0.293	0	7	CDA
0	0.6397699	0.922	0.646	0	7	NCF1
0	0.575064	0.92	0.598	0	7	ALOX5AP
0	0.5606059	0.954	0.736	0	7	NAMPT

0	0.5491029	0.741	0.194	0	7	AC005392.2
0	0.5207235	0.735	0.301	0	7	CMTM2
0	0.5122463	0.848	0.374	0	7	CYSTM1
0	0.5087838	0.72	0.239	0	7	MMP9
0	0.5000326	0.903	0.377	0	7	FPR1
0	0.4863373	0.967	0.616	0	7	LST1
0	0.4832081	0.953	0.451	0	7	MNDA
1.53E-269	0.5011297	0.717	0.337	3.05E-266	7	C5AR1
8.63E-96	0.5057876	0.593	0.548	1.73E-92	7	RGS2
4.23E-62	0.4951909	0.428	0.143	8.47E-59	7	HP
0	0.8607386	0.995	0.969	0	8	IL7R
0	0.8147189	0.984	0.751	0	8	LTB
0	0.6374556	0.832	0.921	0	8	CCR7
0	0.6103427	0.802	0.904	0	8	MAL
0	0.5864525	0.889	0.89	0	8	CAMK4
0	0.5730618	0.96	0.805	0	8	KLF2
0	0.5719437	0.848	0.849	0	8	RCAN3
0	0.5516319	0.79	0.747	0	8	CREM
0	0.548043	0.99	0.947	0	8	LDHB
0	0.5453172	0.896	0.892	0	8	S1PR1
0	0.5366143	0.796	0.851	0	8	SESN3
0	0.5356501	0.851	0.896	0	8	TCF7
0	0.5137812	0.949	0.906	0	8	PDCD4
0	0.5057276	0.986	0.982	0	8	PIK3IP1
0	0.505015	0.97	0.96	0	8	GAS5
0	0.5030173	0.756	0.824	0	8	TRAT1
0	0.4818137	0.847	0.895	0	8	CD5
0	0.4782253	0.862	0.963	0	8	SNX9
0	0.4732219	0.979	0.855	0	8	CD3E
6.90E-249	0.4639076	0.686	0.724	1.38E-245	8	GATA3
0	0.9196455	0.966	0.592	0	9	CD8B
0	0.8810685	0.98	0.565	0	9	CD8A
0	0.8582681	1	0.665	0	9	CCL5
0	0.8010093	0.999	0.558	0	9	NKG7
0	0.7101716	0.954	0.389	0	9	GZMH
0	0.6633117	0.999	0.854	0	9	CD3E
0	0.6472943	0.998	0.75	0	9	IL32
0	0.6346613	0.982	0.539	0	9	TRBV11-2
0	0.6186127	0.973	0.703	0	9	CD3G
0	0.5841526	0.995	0.827	0	9	CD3D
0	0.5632548	0.967	0.795	0	9	CD2
0	0.5444476	0.957	0.735	0	9	GZMM
0	0.5381593	0.964	0.754	0	9	PTPRCAP
0	0.5332627	0.991	0.624	0	9	CST7
0	0.5249561	0.911	0.6	0	9	KLRK1
0	0.5118738	0.968	0.749	0	9	LCK
0	0.5038441	0.996	0.722	0	9	DUSP2
0	0.4973486	0.934	0.837	0	9	CD6
0	0.4965395	0.978	0.685	0	9	JUN
2.46E-94	0.4959242	0.649	0.554	4.93E-91	9	GZMK
0	1.2927473	1	0.692	0	10	LYZ

0	1.2442598	0.996	0.454	0	10	VCAN
0	1.0740725	1	0.603	0	10	S100A9
0	1.0723156	1	0.73	0	10	CST3
0	1.070629	1	0.525	0	10	IFI30
0	1.0489182	0.988	0.424	0	10	GRN
0	1.0473385	0.998	0.543	0	10	FCN1
0	0.9911326	0.976	0.304	0	10	CD14
0	0.9831093	0.988	0.674	0	10	APLP2
0	0.9625927	0.995	0.665	0	10	LGALS3
0	0.9537731	0.996	0.675	0	10	CD68
0	0.9464454	0.998	0.557	0	10	TYMP
0	0.9373915	0.982	0.515	0	10	CSTA
0	0.8909681	0.991	0.449	0	10	S100A12
0	0.8772739	0.998	0.726	0	10	LGALS1
0	0.8732888	1	0.748	0	10	CTSS
0	0.8701085	0.995	0.604	0	10	CEBPD
0	0.8685776	0.993	0.496	0	10	TNFAIP2
0	0.8671754	1	0.566	0	10	S100A8
0	0.850697	0.952	0.482	0	10	LRP1
0	1.0857026	0.979	0.915	0	11	CCR7
0	0.9094988	0.977	0.891	0	11	TCF7
0	0.8812226	0.906	0.891	0	11	LEF1
0	0.8139578	0.992	0.753	0	11	LTB
0	0.8033162	0.843	0.669	0	11	PRKCQ-AS1
0	0.7960085	0.993	0.97	0	11	IL7R
0	0.7805465	0.964	0.887	0	11	CAMK4
0	0.7093723	0.953	0.793	0	11	GIMAP7
0	0.7000465	0.998	0.981	0	11	PIK3IP1
0	0.685186	0.873	0.653	0	11	GIMAP5
0	0.6503216	0.787	0.904	0	11	MAL
0	0.6369605	0.883	0.848	0	11	RCAN3
0	0.635595	0.889	0.842	0	11	SATB1
0	0.6315466	0.995	0.947	0	11	LDHB
0	0.6288158	0.99	0.96	0	11	GAS5
0	0.625847	0.919	0.96	0	11	SNX9
0	0.6161248	0.873	0.581	0	11	NOSIP
0	0.597415	0.756	0.807	0	11	TRABD2A
0	0.589504	0.98	0.859	0	11	C12orf57
0	0.5541429	0.791	0.747	0	11	SH3YL1
0	1.4346236	1	0.57	0	12	S100A8
0	1.3249157	1	0.415	0	12	S100P
0	1.221727	1	0.606	0	12	S100A9
0	1.1869807	0.806	0.305	0	12	CMTM2
0	1.1788842	0.932	0.74	0	12	NAMPT
0	1.1744073	0.999	0.406	0	12	AC011472.2
0	1.1609887	0.979	0.486	0	12	MXD1
0	1.1602426	0.966	0.536	0	12	RGS2
0	1.1437677	0.999	0.383	0	12	TREM1
0	1.1392179	0.956	0.649	0	12	NCF1
0	1.1143817	0.966	0.455	0	12	S100A12
0	1.0435262	0.999	0.884	0	12	IFITM2

0	1.0410709	0.901	0.388	0	12	PROK2
0	1.0146439	0.732	0.384	0	12	BASP1
0	0.9913958	0.811	0.346	0	12	G0S2
0	0.9864073	0.961	0.334	0	12	C5AR1
0	0.9815464	0.995	0.875	0	12	S100A11
0	0.9765029	0.952	0.622	0	12	LST1
0	0.9315933	0.845	0.242	0	12	MMP9
0	0.9119109	0.839	0.606	0	12	ALOX5AP
0	1.3687746	0.898	0.32	0	13	CDKN1C
0	1.2049737	0.998	0.476	0	13	HMOX1
0	1.1526675	1	0.734	0	13	CST3
0	1.1450809	0.992	0.505	0	13	SERPINA1
0	1.1362339	1	0.622	0	13	LST1
0	1.1014154	0.978	0.326	0	13	LILRB2
0	1.0910236	0.983	0.478	0	13	WARS
0	1.0846364	1	0.73	0	13	AIF1
0	1.0811999	1	0.532	0	13	IFI30
0	1.0520157	0.997	0.679	0	13	CD68
0	1.0509889	0.958	0.527	0	13	RHOC
0	1.0444759	0.957	0.4	0	13	LRRC25
0	1.040918	0.973	0.671	0	13	CUX1
0	1.0397424	0.926	0.164	0	13	MS4A7
0	1.035439	0.978	0.539	0	13	HES4
0	1.0349716	0.979	0.44	0	13	CFD
0	1.0318939	0.949	0.492	0	13	PECAM1
0	1.0255169	0.986	0.416	0	13	FCGR3A
0	1.0179046	0.996	0.814	0	13	HLA-DPA1
0	1.0167482	0.99	0.504	0	13	LYN
0	1.0883853	1	0.565	0	14	NKG7
0	1.0323612	0.874	0.539	0	14	GNLY
0	0.9840515	0.998	0.671	0	14	CCL5
0	0.9692203	0.962	0.326	0	14	GZMB
0	0.924183	0.944	0.651	0	14	CTSW
0	0.9104562	0.992	0.63	0	14	CST7
0	0.8455879	0.924	0.389	0	14	FGFBP2
0	0.7919516	0.955	0.504	0	14	PRF1
0	0.7643892	0.946	0.386	0	14	GZMA
0	0.7616666	0.796	0.366	0	14	CCL4
0	0.761446	0.827	0.313	0	14	S1PR5
0	0.7605421	0.868	0.434	0	14	KLRD1
0	0.7392635	0.935	0.4	0	14	GZMH
0	0.7148661	0.937	0.466	0	14	CMC1
0	0.7008025	0.796	0.522	0	14	SPON2
0	0.6974261	0.938	0.739	0	14	GZMM
0	0.6933464	0.83	0.568	0	14	TRBC1
0	0.6803374	0.981	0.755	0	14	IL32
0	0.6218289	0.849	0.789	0	14	KLRB1
0	0.5964746	0.962	0.863	0	14	CD7
0	1.0669425	0.9	0.55	0	15	GZMK
0	0.6850088	0.883	0.677	0	15	CD27
0	0.6459051	0.971	0.728	0	15	DUSP2

0	0.6293352	0.994	0.857	0	15	CD3E
0	0.6020997	0.996	0.906	0	15	ETS1
0	0.5305781	0.978	0.831	0	15	CD3D
0	0.5154662	0.959	0.926	0	15	CLDND1
0	0.5103571	0.918	0.784	0	15	CBLB
0	0.507221	0.965	0.517	0	15	DUSP4
0	0.494455	0.912	0.77	0	15	TRBC2
0	0.4820903	0.999	0.937	0	15	CXCR4
0	0.4792965	0.99	0.91	0	15	RHOH
0	0.4776159	0.974	0.762	0	15	PMAIP1
0	0.4719219	0.957	0.789	0	15	LIME1
0	0.4718609	0.944	0.837	0	15	RGS1
0	0.4670995	0.936	0.66	0	15	SYNE2
3.58E-291	0.5346159	0.825	0.714	7.17E-288	15	ITM2A
5.36E-258	0.5598353	0.826	0.603	1.07E-254	15	CD8B
1.63E-223	0.528006	0.825	0.577	3.26E-220	15	CD8A
6.32E-155	0.4677296	0.66	0.48	1.26E-151	15	TIGIT
0	0.6065009	0.956	0.861	0	16	MTRNR2L12
0	0.5153494	0.99	0.97	0	16	IL7R
0	0.495338	0.944	0.951	0	16	MT-ND4L
0	0.4500571	0.986	0.924	0	16	SPOCK2
0	0.4387677	0.982	0.975	0	16	MT-CYB
0	0.4205491	0.969	0.845	0	16	SESN3
0	0.4098677	0.995	0.971	0	16	MT-CO3
1.07E-307	0.3575941	0.94	0.935	2.14E-304	16	MT-ND3
1.82E-251	0.5277858	0.791	0.764	3.65E-248	16	MT-ATP8
7.48E-225	0.6456368	0.757	0.665	1.50E-221	16	SYNE2
2.15E-189	0.3636894	0.896	0.89	4.31E-186	16	CAMK4
3.79E-176	0.3927346	0.955	0.907	7.59E-173	16	ETS1
1.34E-131	0.4405673	0.842	0.904	2.68E-128	16	ITK
1.87E-114	0.4263212	0.828	0.787	3.73E-111	16	CBLB
7.10E-91	0.3659037	0.814	0.81	1.42E-87	16	FYN
1.19E-82	0.410288	0.771	0.772	2.39E-79	16	BCL11B
1.03E-69	0.4479001	0.523	0.39	2.06E-66	16	SPTAN1
1.39E-69	0.3569276	0.815	0.842	2.77E-66	16	CD6
5.76E-65	0.40105	0.805	0.916	1.15E-61	16	RORA
4.89E-23	0.3737853	0.594	0.644	9.77E-20	16	IKZF3
0	1.3048863	0.796	0.352	0	17	G0S2
0	1.2309634	0.799	0.311	0	17	CMTM2
0	1.1582447	1	0.39	0	17	TREM1
0	1.1250091	0.988	0.575	0	17	S100A8
0	1.1151863	1	0.413	0	17	AC011472.2
0	1.0033108	1	0.802	0	17	NFKBIZ
0	0.9946134	0.944	0.742	0	17	NAMPT
0	0.990456	0.878	0.543	0	17	RGS2
0	0.982961	0.975	0.611	0	17	S100A9
0	0.9798775	1	0.422	0	17	S100P
0	0.9600163	1	0.885	0	17	IFITM2
0	0.9557105	0.93	0.842	0	17	SOD2
0	0.947392	0.905	0.542	0	17	FOS
0	0.9448062	0.924	0.28	0	17	FCGR3B

0	0.8950172	0.89	0.494	0	17	MXD1
0	0.8949282	0.946	0.626	0	17	LST1
1.88E-284	1.103878	0.73	0.175	3.75E-281	17	CXCL8
7.02E-282	0.8876421	0.737	0.255	1.40E-278	17	SMIM25
7.79E-205	1.0811462	0.677	0.39	1.56E-201	17	BASP1
5.47E-41	0.9269664	0.565	0.145	1.09E-37	17	TNFRSF10C
0	0.7132974	0.995	0.575	0	18	S100A8
0	0.5959142	0.951	0.742	0	18	NAMPT
0	0.5664982	0.93	0.842	0	18	SOD2
0	0.4131472	0.9	0.425	0	18	S100P
3.21E-302	0.6066764	0.956	0.733	6.42E-299	18	AIF1
7.87E-292	0.4628527	0.87	0.392	1.57E-288	18	FPR1
7.96E-290	0.4736526	0.992	0.734	1.59E-286	18	IER3
2.54E-257	0.5095728	0.877	0.466	5.08E-254	18	MNDA
1.26E-238	0.637772	0.746	0.353	2.52E-235	18	G0S2
4.89E-208	0.4148296	0.993	0.777	9.78E-205	18	IFITM3
4.32E-179	0.4732661	0.912	0.627	8.64E-176	18	LST1
9.99E-167	0.5581587	0.99	0.612	2.00E-163	18	S100A9
1.57E-162	0.3956975	0.741	0.389	3.14E-159	18	BASP1
2.60E-149	0.5674127	0.79	0.453	5.20E-146	18	MARCKS
4.75E-143	0.3994298	0.803	0.418	9.50E-140	18	AC011472.2
2.23E-137	0.5611658	0.916	0.804	4.45E-134	18	NFKBIZ
2.96E-130	0.4209424	0.942	0.649	5.93E-127	18	SPI1
1.33E-58	0.4823239	0.701	0.513	2.66E-55	18	TNFAIP2
4.34E-45	0.588624	0.78	0.728	8.68E-42	18	IFIT3
7.61E-07	0.4122433	0.471	0.459	0.0015217	18	CSF2RB
0	0.8782722	0.996	0.699	0	19	LYZ
0	0.7798432	0.992	0.736	0	19	CST3
0	0.6825828	0.912	0.468	0	19	VCAN
0	0.6474038	0.89	0.528	0	19	CSTA
0	0.4563033	0.961	0.48	0	19	SCO2
0	0.4537818	0.985	0.845	0	19	IFNGR2
1.86E-269	0.4726744	0.953	0.81	3.72E-266	19	CTSD
3.13E-267	0.543622	0.916	0.684	6.26E-264	19	CD68
3.50E-265	0.5431803	0.969	0.733	6.99E-262	19	LGALS1
2.31E-251	0.637635	0.982	0.537	4.62E-248	19	IFI30
4.42E-247	0.5692307	0.948	0.554	8.85E-244	19	FCN1
3.57E-233	0.4854369	0.904	0.674	7.14E-230	19	LGALS3
1.34E-228	0.6222618	0.989	0.576	2.67E-225	19	S100A8
2.15E-208	0.6193448	0.994	0.612	4.29E-205	19	S100A9
8.41E-201	0.5056446	0.947	0.568	1.68E-197	19	TYMP
3.82E-191	0.4839555	0.877	0.51	7.65E-188	19	TNFAIP2
2.53E-175	0.4670069	0.98	0.733	5.06E-172	19	AIF1
2.99E-168	0.4499017	0.79	0.534	5.99E-165	19	CAPG
7.01E-166	0.4503244	0.849	0.684	1.40E-162	19	APLP2
1.91E-100	0.4760949	0.993	0.711	3.81E-97	19	TYROBP
0	1.4622539	0.93	0.41	0	20	PPBP
0	1.4317005	0.99	0.513	0	20	NRGN
0	1.0351412	0.82	0.325	0	20	TUBB1
0	0.986328	0.839	0.268	0	20	MYL9
0	0.9858905	0.784	0.303	0	20	PF4

0	0.9415777	0.974	0.7	0	20	LYZ
0	0.9279122	0.833	0.437	0	20	CLU
0	0.9043211	0.801	0.35	0	20	PRKAR2B
0	0.9031404	0.981	0.737	0	20	CST3
0	0.8742554	0.96	0.538	0	20	IFI30
0	0.8560075	0.779	0.291	0	20	SPARC
0	0.826696	0.768	0.315	0	20	MPIG6B
0	0.7879123	0.949	0.555	0	20	FCN1
0	0.7646161	0.961	0.683	0	20	CD68
0	0.7595912	0.944	0.744	0	20	HLA-DRA
0	0.72932	0.943	0.569	0	20	TYMP
0	0.7284638	0.957	0.673	0	20	LGALS3
0	0.7032545	0.89	0.439	0	20	GRN
9.92E-244	0.7034904	0.697	0.211	1.98E-240	20	ITGA2B
4.29E-229	0.703481	0.694	0.181	8.57E-226	20	F13A1
0	1.2533116	0.789	0.257	0	21	SMIM25
0	1.2049716	0.793	0.178	0	21	CXCL8
0	1.1876168	1	0.417	0	21	AC011472.2
0	1.183834	1	0.394	0	21	TREM1
0	1.1787237	0.818	0.348	0	21	C5AR1
0	1.1551449	0.997	0.627	0	21	LST1
0	1.0878762	0.963	0.743	0	21	NAMPT
0	1.042321	0.997	0.543	0	21	RGS2
0	1.0329644	1	0.426	0	21	S100P
0	0.9966456	0.964	0.842	0	21	SOD2
0	0.9036656	0.996	0.728	0	21	RAB11FIP1
0	0.9029408	1	0.578	0	21	S100A8
0	0.8822899	0.997	0.614	0	21	S100A9
0	0.8596058	0.985	0.463	0	21	S100A12
0	0.8564474	0.999	0.686	0	21	TXN
5.97E-291	0.9154557	0.817	0.487	1.19E-287	21	BCL2A1
3.74E-276	1.0397182	0.772	0.315	7.47E-273	21	CMTM2
2.13E-244	0.8946685	0.977	0.725	4.26E-241	21	IFIT3
8.08E-103	1.0529492	0.662	0.427	1.62E-99	21	PHACTR1
3.09E-13	0.8776667	0.543	0.359	6.18E-10	21	G0S2
0	1.1216305	1	0.738	0	22	CST3
0	1.0927536	0.999	0.538	0	22	IFI30
0	1.0690109	0.999	0.744	0	22	HLA-DRA
0	1.0346932	1	0.701	0	22	LYZ
0	0.9345335	0.999	0.733	0	22	AIF1
0	0.8903707	0.999	0.815	0	22	HLA-DRB1
0	0.888533	0.995	0.785	0	22	HLA-DRB5
0	0.881246	0.99	0.569	0	22	TYMP
0	0.8721753	0.999	0.817	0	22	HLA-DPA1
0	0.8666022	0.983	0.647	0	22	NPC2
0	0.8625912	0.988	0.683	0	22	CD68
0	0.8623473	0.861	0.366	0	22	LGALS2
0	0.8502972	0.997	0.807	0	22	HLA-DPB1
0	0.8452451	0.968	0.674	0	22	LGALS3
0	0.8405762	0.994	0.555	0	22	FCN1
0	0.833149	0.903	0.55	0	22	CPVL

0	0.8215366	0.974	0.512	0	22	SERPINA1
0	0.8174292	0.972	0.528	0	22	CSTA
0	0.8128127	1	0.713	0	22	TYROBP
0	0.7766931	0.901	0.449	0	22	CFD
0	1.2752452	0.936	0.198	0	23	STMN1
0	0.9982962	0.866	0.149	0	23	MKI67
0	0.9486247	0.906	0.554	0	23	HMGB2
0	0.8413662	0.997	0.818	0	23	HMGN2
0	0.8352402	0.852	0.411	0	23	CENPF
0	0.7813274	0.707	0.075	0	23	BIRC5
0	0.7720151	0.542	0.013	0	23	CDC20
0	0.7356975	0.979	0.776	0	23	H2AFV
0	0.6827977	0.891	0.623	0	23	ANP32E
0	0.6812261	0.969	0.685	0	23	H2AFZ
0	0.6458589	0.892	0.62	0	23	LSM5
0	0.625946	0.985	0.757	0	23	IL32
2.70E-306	0.5806326	0.999	0.865	5.41E-303	23	RAD21
4.09E-306	0.8969697	0.805	0.507	8.18E-303	23	PTTG1
2.47E-303	0.6286278	0.921	0.484	4.94E-300	23	CDK2AP2
2.00E-258	0.7297166	0.931	0.609	4.00E-255	23	TUBA1B
8.86E-144	0.6135633	0.47	0.062	1.77E-140	23	CCNB2
3.64E-119	0.592193	0.697	0.388	7.29E-116	23	UBE2S
7.52E-59	0.6047459	0.531	0.183	1.50E-55	23	CDKN3
2.63E-54	0.6546361	0.582	0.328	5.27E-51	23	TPX2
0	1.0390257	0.977	0.917	0	24	CCR7
0	0.9401289	0.925	0.836	0	24	NELL2
0	0.77971	0.959	0.893	0	24	TCF7
0	0.7687244	0.956	0.889	0	24	CAMK4
0	0.7456171	0.861	0.892	0	24	LEF1
0	0.721888	0.839	0.673	0	24	PRKCQ-AS1
0	0.6786636	0.945	0.958	0	24	SNX9
0	0.6510356	0.99	0.97	0	24	IL7R
0	0.6393655	0.998	0.982	0	24	PIK3IP1
0	0.6268829	0.973	0.907	0	24	PDCD4
0	0.6237295	0.995	0.96	0	24	GAS5
0	0.5764078	0.996	0.948	0	24	LDHB
0	0.5734296	0.985	0.862	0	24	C12orf57
2.89E-307	0.6727504	0.874	0.587	5.78E-304	24	NOSIP
2.80E-285	0.8768196	0.709	0.337	5.60E-282	24	LINC02446
1.81E-281	0.6234435	0.974	0.759	3.63E-278	24	LTB
1.20E-253	0.5792373	0.877	0.843	2.40E-250	24	SATB1
1.97E-232	0.689028	0.857	0.605	3.94E-229	24	CD8B
1.34E-214	0.5868572	0.834	0.611	2.69E-211	24	KLRK1
1.52E-204	0.6001761	0.762	0.805	3.03E-201	24	TRABD2A
0	1.1856278	1	0.579	0	25	S100A8
0	1.0174304	0.888	0.466	0	25	S100A12
0	0.9826375	0.999	0.615	0	25	S100A9
0	0.9708073	0.999	0.427	0	25	S100P
0	0.9015109	0.966	0.743	0	25	NAMPT
0	0.8509551	0.887	0.314	0	25	CMTM2
0	0.7937653	0.999	0.419	0	25	AC011472.2

0	0.7506836	0.955	0.655	0	25	NCF1
0	0.6950935	1	0.878	0	25	S100A11
0	0.6512095	0.998	0.396	0	25	TREM1
0	0.6471402	0.999	0.886	0	25	IFITM2
3.06E-276	0.7366079	0.995	0.728	6.11E-273	25	RAB11FIP1
2.54E-259	0.647469	0.907	0.469	5.08E-256	25	MNDA
3.50E-199	0.7099694	0.793	0.391	6.99E-196	25	BASP1
2.17E-196	0.6825029	0.808	0.536	4.34E-193	25	GCA
1.04E-183	0.7170089	0.803	0.611	2.08E-180	25	ALOX5AP
1.06E-145	0.74291	0.747	0.313	2.13E-142	25	CDA
3.18E-89	0.7792099	0.658	0.49	6.37E-86	25	BCL2A1
2.84E-63	0.6945087	0.67	0.549	5.68E-60	25	RGS2
4.93E-58	0.8627175	0.607	0.403	9.86E-55	25	PROK2
0	1.2116265	0.921	0.2	0	26	STMN1
0	1.1262282	0.979	0.609	0	26	TUBA1B
0	1.0921569	0.869	0.056	0	26	TYMS
0	1.0340004	0.946	0.534	0	26	MCM7
0	0.9568711	0.951	0.428	0	26	MCM5
0	0.9336371	0.946	0.333	0	26	NASP
0	0.9191286	0.921	0.49	0	26	DUT
0	0.8408722	0.953	0.213	0	26	E2F1
0	0.8407663	0.875	0.158	0	26	PCNA
0	0.833059	0.806	0.072	0	26	PCLAF
0	0.8202828	0.964	0.554	0	26	HMGB2
0	0.8180666	0.929	0.439	0	26	DNMT1
0	0.779615	0.898	0.238	0	26	MCM3
0	0.7700384	0.842	0.347	0	26	HELLS
0	0.7673004	0.93	0.394	0	26	GZMA
0	0.7627308	0.972	0.663	0	26	IDH2
0	0.7620452	0.981	0.827	0	26	TUBB
0	0.7607466	0.805	0.052	0	26	GINS2
0	0.7481841	0.925	0.559	0	26	RANBP1
0	0.7355744	0.882	0.268	0	26	CDCA7
0	2.165374	0.922	0.173	0	27	CAMP
0	2.068932	0.909	0.038	0	27	LCN2
0	1.64107	0.923	0.139	0	27	PGLYRP1
0	1.6173541	0.981	0.148	0	27	HP
0	1.5949312	0.996	0.429	0	27	S100P
0	1.5210683	1	0.58	0	27	S100A8
0	1.3689861	0.876	0.467	0	27	S100A12
0	1.1411206	0.996	0.616	0	27	S100A9
0	1.0568847	0.893	0.081	0	27	ORM1
0	1.0247075	0.995	0.702	0	27	LYZ
0	0.9761652	1	0.42	0	27	AC011472.2
0	0.9634853	0.995	0.438	0	27	TPM1
0	0.9184413	0.955	0.726	0	27	TSPO
1.30E-301	1.7416768	0.796	0.224	2.59E-298	27	RETN
7.97E-255	1.2340984	0.804	0.357	1.59E-251	27	PADI4
2.26E-192	0.9479118	0.779	0.313	4.51E-189	27	CDA
3.17E-169	0.8998356	0.775	0.537	6.33E-166	27	GCA
7.91E-151	0.9158389	0.766	0.393	1.58E-147	27	CYSTM1

7.42E-28	1.0617977	0.59	0.259	1.48E-24	27	MMP9
3.28E-11	1.0520927	0.516	0.028	6.55E-08	27	CD24
0	1.511749	0.942	0.544	0	28	GNLY
0	1.2501394	0.97	0.474	0	28	CMC1
0	1.2350399	0.929	0.33	0	28	KLRC1
0	1.1789337	0.988	0.656	0	28	CTSW
0	1.1721933	0.999	0.573	0	28	NKG7
0	1.1597803	0.963	0.44	0	28	KLRD1
0	1.1172786	0.936	0.332	0	28	KLRF1
0	1.1161073	0.993	0.789	0	28	KLRB1
0	1.0891712	0.929	0.39	0	28	IL2RB
0	1.0437629	0.99	0.865	0	28	CD7
0	1.0406354	0.969	0.189	0	28	XCL1
0	1.0333121	0.965	0.395	0	28	GZMA
0	0.9612046	0.994	0.511	0	28	PRF1
0	0.9538321	0.916	0.492	0	28	MATK
0	0.799355	1	0.917	0	28	CD69
5.37E-291	1.0834163	0.924	0.337	1.07E-287	28	GZMB
6.89E-273	0.8229812	0.989	0.637	1.38E-269	28	CST7
5.78E-269	1.1985473	0.874	0.556	1.16E-265	28	GZMK
8.66E-159	0.7896368	0.745	0.203	1.73E-155	28	TRDC
3.48E-158	0.8638609	0.71	0.163	6.96E-155	28	XCL2
0	1.2864511	1	0.581	0	29	S100A8
0	1.2257392	0.998	0.43	0	29	S100P
0	1.1319892	1	0.617	0	29	S100A9
0	1.0608042	0.962	0.745	0	29	NAMPT
0	1.0512201	0.985	0.657	0	29	NCF1
0	1.003688	1	0.422	0	29	AC011472.2
0	0.9493651	1	0.399	0	29	TREM1
0	0.8677948	0.995	0.878	0	29	S100A11
2.44E-299	0.983325	0.938	0.357	4.88E-296	29	G0S2
1.69E-287	0.9682941	0.985	0.547	3.39E-284	29	RGS2
4.63E-282	0.9470135	0.944	0.537	9.25E-279	29	GCA
1.94E-264	0.899479	0.967	0.652	3.87E-261	29	SPI1
4.82E-257	0.9940341	0.974	0.468	9.63E-254	29	S100A12
2.61E-248	0.9318831	0.895	0.398	5.23E-245	29	FPR1
4.55E-245	0.8773647	0.993	0.63	9.09E-242	29	LST1
1.38E-236	0.8825705	0.968	0.499	2.75E-233	29	MXD1
1.27E-178	0.8655557	0.837	0.612	2.54E-175	29	ALOX5AP
5.63E-176	1.0908391	0.815	0.318	1.13E-172	29	CMTM2
1.79E-86	0.8344887	0.723	0.281	3.58E-83	29	DYSF
2.34E-31	0.8664341	0.625	0.403	4.67E-28	29	PROK2
5.91E-225	0.9947491	0.981	0.581	1.18E-221	30	S100A8
1.34E-151	0.4508079	0.992	0.887	2.67E-148	30	IFITM2
2.09E-88	0.6444251	0.971	0.617	4.18E-85	30	S100A9
3.18E-78	0.3921395	0.957	0.863	6.35E-75	30	C12orf57
1.70E-75	0.4017257	0.925	0.834	3.41E-72	30	CD3D
2.69E-69	0.5827465	0.817	0.469	5.38E-66	30	S100A12
1.43E-63	0.6765234	0.651	0.292	2.87E-60	30	FCGR3B
8.84E-55	0.4398314	0.81	0.746	1.77E-51	30	NAMPT
4.55E-49	0.3710664	0.917	0.843	9.10E-46	30	SOD2

1.05E-48	0.3705837	0.825	0.773	2.10E-45	30	TRBC2
2.22E-45	0.4176177	0.779	0.473	4.45E-42	30	MNDA
4.53E-38	0.489632	0.616	0.36	9.06E-35	30	G0S2
2.09E-33	0.408032	0.69	0.4	4.18E-30	30	FPR1
1.11E-27	0.4007929	0.622	0.316	2.22E-24	30	CDA
1.31E-24	0.4003554	0.607	0.32	2.63E-21	30	CMTM2
5.67E-22	0.3693123	0.645	0.539	1.13E-18	30	GCA
8.06E-18	0.445716	0.466	0.181	1.61E-14	30	AQP9
3.81E-14	0.409089	0.496	0.255	7.63E-11	30	MMP25
4.99E-13	0.3611614	0.448	0.299	9.99E-10	30	VNN2
2.66E-11	0.4094163	0.452	0.113	5.32E-08	30	ALPL
0	1.0869588	0.994	0.831	0	31	NEAT1
1.41E-254	1.0960269	0.97	0.514	2.81E-251	31	TNFAIP2
9.20E-253	1.0108309	0.974	0.452	1.84E-249	31	ADGRE2
7.64E-234	1.0166644	0.929	0.475	1.53E-230	31	PLXDC2
7.18E-229	1.0073156	0.968	0.52	1.44E-225	31	CSF3R
2.51E-221	0.9369428	0.986	0.778	5.02E-218	31	SLC25A37
1.54E-205	0.8003902	0.983	0.659	3.07E-202	31	ZMIZ1
1.25E-195	0.9379708	0.935	0.493	2.49E-192	31	SLC11A1
3.00E-190	0.8615984	0.946	0.71	5.99E-187	31	ETV6
2.41E-189	0.9810078	0.892	0.5	4.83E-186	31	LRP1
1.17E-183	0.8846586	0.931	0.558	2.35E-180	31	IL17RA
2.61E-173	0.8840176	0.91	0.501	5.22E-170	31	POU2F2
1.05E-160	0.8121792	0.908	0.437	2.09E-157	31	CLEC7A
1.13E-158	0.8591764	0.901	0.637	2.26E-155	31	GNAQ
1.76E-137	0.8707342	0.845	0.405	3.53E-134	31	ALOX5
3.06E-121	0.7842037	0.92	0.551	6.13E-118	31	FGR
2.40E-109	0.8248997	0.839	0.713	4.81E-106	31	DENND5A
2.77E-102	0.8938925	0.792	0.475	5.53E-99	31	VCAN
1.24E-96	0.9362704	0.742	0.454	2.48E-93	31	DMXL2
8.83E-83	0.7669853	0.758	0.413	1.77E-79	31	CD300E
1.73E-157	0.3998747	0.886	0.218	3.46E-154	32	HCAR2
6.21E-129	0.6694385	0.971	0.52	1.24E-125	32	CSF3R
2.36E-112	0.5458446	0.94	0.745	4.73E-109	32	NAMPT
3.51E-111	0.7941425	0.27	0.101	7.03E-108	32	PI3
5.32E-71	0.4513863	0.983	0.831	1.06E-67	32	NEAT1
1.75E-52	0.7970909	0.326	0.14	3.51E-49	32	SLPI
2.93E-51	0.5907326	0.844	0.806	5.85E-48	32	NFKBIZ
9.36E-46	0.4029282	0.76	0.757	1.87E-42	32	RNASET2
1.84E-41	0.4082625	0.25	0.226	3.67E-38	32	SIGLEC10
3.62E-39	0.4743669	0.732	0.377	7.25E-36	32	ITGAX
3.10E-36	0.5948309	0.745	0.516	6.21E-33	32	TNFAIP2
8.94E-27	0.421442	0.673	0.681	1.79E-23	32	PPP1R15A
9.70E-24	0.556913	0.706	0.402	1.94E-20	32	TREM1
9.09E-18	0.4578022	0.272	0.112	1.82E-14	32	CYP27A1
5.61E-13	0.4274864	0.698	0.732	1.12E-09	32	RAB11FIP1
1.03E-11	0.4053937	0.628	0.429	2.05E-08	32	PHACTR1
2.92E-08	0.4474643	0.668	0.674	5.85E-05	32	ABCA1
4.42E-06	0.4698758	0.416	0.221	0.0088329	32	LUCAT1
1.71E-05	0.6838298	0.537	0.361	0.0342701	32	G0S2
0.0016631	0.5989554	0.448	0.55	1	32	CXCL16

0	1.3788081	1	0.747	0	33	HLA-DRA
0	0.8552134	1	0.957	0	33	CD74
1.27E-307	1.1620647	1	0.817	2.54E-304	33	HLA-DRB1
3.35E-306	1.234062	1	0.809	6.71E-303	33	HLA-DPB1
4.13E-298	1.2007012	1	0.787	8.27E-295	33	HLA-DRB5
3.06E-296	1.1752182	1	0.819	6.12E-293	33	HLA-DPA1
1.90E-291	1.2053762	1	0.705	3.80E-288	33	HLA-DQB1
1.07E-284	1.4534997	0.994	0.409	2.13E-281	33	HLA-DQA1
6.49E-277	1.017079	0.986	0.605	1.30E-273	33	HLA-DQA2
2.12E-273	1.208618	1	0.74	4.23E-270	33	CST3
4.45E-265	1.0516676	1	0.595	8.89E-262	33	HLA-DMA
1.94E-238	1.0370184	0.986	0.553	3.89E-235	33	CPVL
1.39E-235	0.9027319	1	0.721	2.79E-232	33	ANXA2
4.47E-221	0.9123451	0.957	0.447	8.93E-218	33	HLA-DMB
1.36E-215	0.8585698	0.994	0.622	2.73E-212	33	ZNF385A
1.11E-206	0.8574801	1	0.736	2.22E-203	33	LGALS1
2.35E-203	0.881777	0.994	0.472	4.69E-200	33	YWHAH
1.39E-187	0.9492071	1	0.445	2.77E-184	33	GRN
2.07E-160	1.0376815	1	0.704	4.14E-157	33	LYZ
5.83E-143	0.9171441	1	0.543	1.17E-139	33	IFI30
0	1.7008973	0.975	0.062	0	34	TYMS
0	1.6180082	0.978	0.077	0	34	RRM2
0	1.3888769	0.966	0.225	0	34	UBE2C
0	1.2235357	0.978	0.078	0	34	CCNA2
5.85E-305	1.2676707	0.96	0.077	1.17E-301	34	PCLAF
1.03E-288	1.4208987	0.982	0.156	2.07E-285	34	MKI67
1.78E-284	1.6037374	1	0.612	3.55E-281	34	TUBA1B
9.64E-284	1.8275387	0.991	0.205	1.93E-280	34	STMN1
1.07E-283	1.2534262	0.966	0.123	2.14E-280	34	CENPM
3.28E-283	1.340639	1	0.828	6.56E-280	34	TUBB
1.36E-270	1.4939223	0.996	0.557	2.72E-267	34	HMGB2
6.81E-263	1.6239671	0.991	0.588	1.36E-259	34	HIST1H4C
6.81E-257	1.4028521	0.971	0.304	1.36E-253	34	NUSAP1
9.85E-235	1.2758945	0.969	0.493	1.97E-231	34	DUT
2.18E-230	1.229787	0.951	0.164	4.36E-227	34	PCNA
9.00E-223	1.3567141	0.975	0.375	1.80E-219	34	CCL4
2.37E-218	1.2324112	0.953	0.495	4.74E-215	34	HIST1H3D
1.24E-215	1.2636438	0.919	0.075	2.49E-212	34	HIST1H1B
1.23E-207	1.1910518	0.957	0.398	2.46E-204	34	GZMA
1.57E-206	1.1899672	0.953	0.39	3.14E-203	34	UBE2S
6.27E-193	0.716711	0.995	0.938	1.25E-189	35	CXCR4
1.07E-173	0.7113973	0.995	0.96	2.15E-170	35	TNFAIP3
1.95E-170	0.8254106	0.931	0.85	3.90E-167	35	ZNF331
1.97E-166	1.0068404	0.896	0.683	3.95E-163	35	NR4A2
1.94E-165	0.9467697	0.944	0.733	3.88E-162	35	DUSP2
6.05E-164	0.8011703	0.988	0.925	1.21E-160	35	SPOCK2
1.06E-140	0.6819347	0.986	0.92	2.12E-137	35	ARL4C
1.13E-139	0.7415584	0.919	0.77	2.25E-136	35	PDE4B
3.46E-137	0.8274097	0.894	0.748	6.93E-134	35	CREM
5.37E-134	0.7004185	0.979	0.918	1.07E-130	35	CD69
7.91E-126	0.9619971	0.831	0.557	1.58E-122	35	GZMK

3.59E-118	0.7042038	0.866	0.745	7.19E-115	35	PDE4D
3.31E-113	0.6647606	0.938	0.913	6.62E-110	35	RORA
4.09E-103	0.6933782	0.854	0.821	8.19E-100	35	TSPYL2
2.21E-95	0.7723509	0.979	0.678	4.41E-92	35	CCL5
1.43E-90	0.752331	0.9	0.697	2.86E-87	35	JUN
6.04E-85	0.6753553	0.794	0.587	1.21E-81	35	LYAR
1.30E-84	0.7432242	0.845	0.582	2.60E-81	35	CD8A
6.38E-82	0.8427267	0.794	0.831	1.28E-78	35	CCR6
3.18E-69	0.962457	0.822	0.791	6.36E-66	35	KLRB1
1.16E-105	0.8072042	0.939	0.476	2.31E-102	36	VCAN
1.25E-92	0.6138799	0.995	0.832	2.51E-89	36	NEAT1
3.45E-85	0.8679655	0.98	0.704	6.89E-82	36	LYZ
2.03E-79	0.7188352	0.977	0.561	4.06E-76	36	FCN1
4.96E-79	0.6308524	0.982	0.758	9.91E-76	36	CTSS
1.62E-78	0.6221344	0.965	0.686	3.24E-75	36	APLP2
5.21E-78	0.7916732	0.992	0.618	1.04E-74	36	S100A9
5.85E-74	0.5824956	0.884	0.368	1.17E-70	36	MS4A6A
6.23E-72	0.674358	0.965	0.574	1.25E-68	36	TYMP
1.31E-69	0.5713694	0.97	0.515	2.62E-66	36	TNFAIP2
4.71E-69	0.6572135	0.856	0.331	9.43E-66	36	CD14
5.15E-69	0.5656218	0.924	0.534	1.03E-65	36	CSTA
2.51E-66	0.5530891	0.962	0.779	5.02E-63	36	SLC25A37
1.37E-65	0.7217716	0.985	0.741	2.75E-62	36	CST3
8.83E-65	0.6349587	0.901	0.446	1.77E-61	36	GRN
2.26E-64	0.5507402	0.944	0.678	4.52E-61	36	LGALS3
5.13E-64	0.704878	0.967	0.544	1.03E-60	36	IFI30
1.53E-61	0.7214725	0.982	0.583	3.06E-58	36	S100A8
2.70E-47	0.5490008	0.977	0.737	5.41E-44	36	AIF1
4.62E-41	0.5569886	0.977	0.716	9.24E-38	36	TYROBP
9.61E-204	1.7903893	0.976	0.23	1.92E-200	37	C2orf88
3.53E-183	2.0070968	0.984	0.189	7.05E-180	37	F13A1
2.20E-182	2.1291806	1	0.299	4.39E-179	37	SPARC
1.63E-180	2.2072012	1	0.311	3.27E-177	37	PF4
8.54E-180	2.3298449	1	0.333	1.71E-176	37	TUBB1
1.04E-169	2.3408544	1	0.521	2.09E-166	37	NRGN
1.09E-167	2.4249777	0.996	0.419	2.18E-164	37	PPBP
9.54E-166	1.8769416	0.972	0.287	1.91E-162	37	PTGS1
1.13E-164	2.0071529	0.98	0.357	2.26E-161	37	PRKAR2B
9.69E-160	1.7771829	0.988	0.39	1.94E-156	37	AP003068.2
3.02E-159	1.765355	0.988	0.255	6.04E-156	37	TSPAN33
5.19E-157	1.8171307	0.961	0.361	1.04E-153	37	ACRBP
5.05E-155	2.0398441	0.984	0.443	1.01E-151	37	CLU
1.61E-153	1.7896151	0.984	0.478	3.22E-150	37	LIMS1
6.34E-152	2.0946468	0.953	0.322	1.27E-148	37	MPIG6B
1.16E-151	1.7790175	0.957	0.464	2.33E-148	37	TRIM58
3.12E-149	2.062251	0.953	0.277	6.23E-146	37	MYL9
1.31E-142	1.8585641	0.957	0.304	2.61E-139	37	HIST1H2AC
1.24E-140	1.841597	0.949	0.233	2.48E-137	37	RGS18
1.03E-134	1.8397993	0.898	0.218	2.06E-131	37	ITGA2B
1.13E-39	1.227785	0.95	0.763	2.26E-36	38	ITM2C
8.11E-37	0.8886443	0.967	0.76	1.62E-33	38	APP

1.64E-30	0.9050156	0.595	0.115	3.28E-27	38	TCF4
1.56E-27	0.7389851	0.405	0.021	3.12E-24	38	DERL3
2.13E-25	0.7484093	0.917	0.7	4.27E-22	38	SPINT2
1.69E-24	0.7618563	0.843	0.652	3.39E-21	38	C12orf75
2.82E-23	0.8453736	0.802	0.342	5.63E-20	38	GZMB
2.41E-21	0.970905	0.62	0.313	4.81E-18	38	SERPINF1
2.43E-21	0.9411455	0.893	0.797	4.86E-18	38	PPP1R14B
2.80E-21	0.835107	0.587	0.2	5.60E-18	38	MAP1A
6.35E-20	0.9412385	0.752	0.431	1.27E-16	38	IRF7
2.80E-19	0.7093302	0.653	0.208	5.61E-16	38	STMN1
1.35E-18	0.8897228	0.777	0.466	2.70E-15	38	TPM2
1.20E-17	0.7065983	0.488	0.097	2.41E-14	38	MZB1
2.08E-17	0.8460913	0.868	0.645	4.15E-14	38	CCDC50
2.84E-17	0.6947814	0.76	0.542	5.67E-14	38	CXXC5
5.41E-17	0.8109597	0.545	0.271	1.08E-13	38	IL3RA
6.38E-17	0.747539	0.628	0.375	1.28E-13	38	BCL11A
9.45E-11	0.820785	0.868	0.75	1.89E-07	38	IRF8
7.59E-08	0.7298778	0.711	0.522	0.0001518	38	NRGN
1.68E-29	0.3039885	0.991	0.972	3.37E-26	39	MT-CO3
9.52E-28	0.7026096	0.945	0.863	1.90E-24	39	MTRNR2L12
6.22E-26	0.6422583	0.9	0.958	1.24E-22	39	AL138963.4
7.75E-25	0.4800045	0.945	0.951	1.55E-21	39	MT-ND4L
8.93E-20	0.9946242	0.6	0.42	1.79E-16	39	PPBP
6.72E-19	0.4778841	0.891	0.884	1.34E-15	39	AHNAK
3.11E-15	0.669076	0.955	0.705	6.22E-12	39	LYZ
4.02E-15	0.3211173	0.864	0.95	8.04E-12	39	MT-ND2
1.43E-14	0.3387738	0.855	0.935	2.85E-11	39	MT-ND3
1.15E-10	0.5183835	0.945	0.584	2.30E-07	39	S100A8
6.91E-10	0.7778477	0.564	0.522	1.38E-06	39	NRGN
7.39E-10	0.5086789	0.955	0.619	1.48E-06	39	S100A9
2.30E-08	0.3329862	0.3	0.376	4.61E-05	39	MTRNR2L8
6.15E-08	0.3639688	0.673	0.857	0.0001229	39	MT-ND5
6.20E-06	0.4027697	0.291	0.279	0.0123915	39	MYL9
4.03E-05	0.4415308	0.318	0.445	0.0805209	39	CLU
0.0006833	0.6066214	0.309	0.313	1	39	PF4
0.001572	0.5825341	0.336	0.334	1	39	TUBB1
0.0021341	0.3766221	0.236	0.465	1	39	TRIM58
0.0058376	0.3353644	0.518	0.662	1	39	ZMIZ1
2.99E-83	2.3589589	0.958	0.123	5.98E-80	40	CLC
6.80E-57	1.2339743	1	0.728	1.36E-53	40	AREG
7.68E-57	0.8553974	0.927	0.265	1.54E-53	40	MS4A4E
4.61E-49	1.6858635	0.927	0.459	9.21E-46	40	CSF2RB
5.93E-49	1.1044469	0.927	0.123	1.19E-45	40	BTK
5.03E-44	0.9519941	0.969	0.751	1.01E-40	40	SWAP70
1.85E-43	1.2881468	0.979	0.499	3.70E-40	40	PGD
4.08E-42	0.9691231	0.969	0.734	8.15E-39	40	HPGD
2.65E-41	1.0542868	0.958	0.256	5.31E-38	40	MMP25
2.96E-40	1.0727106	0.958	0.308	5.92E-37	40	RAB32
5.62E-37	1.0744271	0.979	0.521	1.12E-33	40	FAM110A
5.88E-33	1.3367092	0.865	0.456	1.18E-29	40	CFD
6.21E-33	1.1515664	0.948	0.614	1.24E-29	40	ALOX5AP

3.33E-26	0.8600974	0.938	0.453	6.65E-23	40	AL627171.2
1.94E-22	0.8618287	0.948	0.537	3.89E-19	40	GLUL
1.59E-20	0.9427182	0.875	0.55	3.19E-17	40	RGS2
1.43E-18	0.8495693	0.875	0.559	2.87E-15	40	HMGB2
9.68E-07	1.0493775	0.615	0.22	0.0019356	40	ITGA2B
0.0003253	1.0647013	0.573	0.271	0.6506905	40	IL3RA
0.0040858	0.880471	0.542	0.35	1	40	CR1
6.88E-46	1.3023225	0.966	0.746	1.38E-42	41	NAMPT
4.49E-43	1.0950279	0.989	0.403	8.98E-40	41	TREM1
6.77E-43	1.1712554	0.955	0.522	1.35E-39	41	CSF3R
6.04E-39	0.9573792	0.966	0.832	1.21E-35	41	NEAT1
3.23E-27	1.1102386	0.921	0.503	6.45E-24	41	MXD1
1.56E-24	0.9457497	0.955	0.732	3.12E-21	41	RAB11FIP1
1.74E-24	0.9096	0.82	0.229	3.47E-21	41	ADGRG3
2.67E-22	0.8448314	0.955	0.807	5.33E-19	41	NFKBIZ
1.29E-19	0.8746907	0.809	0.288	2.57E-16	41	P2RX1
2.17E-19	0.8858969	0.742	0.139	4.34E-16	41	MGAM
1.40E-17	1.0403602	0.809	0.674	2.80E-14	41	ABCA1
6.50E-16	0.8443601	0.809	0.386	1.30E-12	41	RBM47
1.42E-10	1.0650366	0.674	0.284	2.85E-07	41	DYSF
8.06E-10	0.9727109	0.652	0.379	1.61E-06	41	ITGAX
2.04E-08	1.0165819	0.64	0.222	4.08E-05	41	LUCAT1
2.51E-08	0.9111905	0.64	0.257	5.01E-05	41	LRRK2
4.84E-06	0.9307698	0.596	0.183	0.0096785	41	AQP9
2.55E-05	1.0578484	0.551	0.117	0.051091	41	KCNJ15
8.29E-05	1.0359975	0.562	0.224	0.1657904	41	CREB5
0.0003132	1.0467097	0.427	0.017	0.6263683	41	EPHB1

Supp. table 2.

ID	scTCR	scRNA	share Freq
KXD1	1340	1445	93%
KXD2	1432	1860	77%
KXD3	4960	6035	82%
LP1	5197	6504	80%
LP2	1794	2354	76%
LP3	212	284	75%
LP4	620	756	82%
SXH1	4139	8745	47%
SXH2	4813	6448	75%
XSR1	3274	3489	94%
XSR2	313	335	93%
XSR3	4061	4687	87%
ZRH1	1115	1300	86%
ZRH2	3419	4573	75%
ZRH3	1799	2775	65%
	38488	51590	

Table 3

ID	clonetype	clonality
KXD1	886	0.08473238
KXD2	710	0.156617958
KXD3	492	0.365468939
LP1	4893	0.007887083
LP2	1753	0.001445187
LP3	212	1.11022E-16
LP4	572	0.009889195
SXH1	1562	0.267451332
SXH2	2154	0.117503186
XSR1	535	0.383462405
XSR2	161	0.157027483
XSR3	420	0.371245873
ZRH1	838	0.064517607
ZRH2	676	0.286081024
ZRH3	550	0.222791269
